# Conserved novel ORFs in the mitochondrial genome of the ctenophore *Beroe forskalii*

**DOI:** 10.7717/peerj.8356

**Published:** 2020-01-27

**Authors:** Darrin T. Schultz, Jordan M. Eizenga, Russell B. Corbett-Detig, Warren R. Francis, Lynne M. Christianson, Steven H.D. Haddock

**Affiliations:** 1 Department of Biomolecular Engineering and Bioinformatics, University of California Santa Cruz, Santa Cruz, CA, USA; 2 Monterey Bay Aquarium Research Institute, Moss Landing, CA, USA; 3 Department of Biology, University of Southern Denmark, Odense, Denmark; 4 Department of Ecology and Evolutionary Biology, University of California Santa Cruz, Santa Cruz, CA, USA

**Keywords:** Ctenophore, Mitochondria, Mitogenome, Selection, Evolution, Bioinformatics, URF, ORF, Bayesian, Sequencing

## Abstract

To date, five ctenophore species’ mitochondrial genomes have been sequenced, and each contains open reading frames (ORFs) that if translated have no identifiable orthologs. ORFs with no identifiable orthologs are called unidentified reading frames (URFs). If truly protein-coding, ctenophore mitochondrial URFs represent a little understood path in early-diverging metazoan mitochondrial evolution and metabolism. We sequenced and annotated the mitochondrial genomes of three individuals of the beroid ctenophore *Beroe forskalii* and found that in addition to sharing the same canonical mitochondrial genes as other ctenophores, the *B. forskalii* mitochondrial genome contains two URFs. These URFs are conserved among the three individuals but not found in other sequenced species. We developed computational tools called pauvre and cuttlery to determine the likelihood that URFs are protein coding. There is evidence that the two URFs are under negative selection, and a novel Bayesian hypothesis test of trinucleotide frequency shows that the URFs are more similar to known coding genes than noncoding intergenic sequence. Protein structure and function prediction of all ctenophore URFs suggests that they all code for transmembrane transport proteins. These findings, along with the presence of URFs in other sequenced ctenophore mitochondrial genomes, suggest that ctenophores may have uncharacterized transmembrane proteins present in their mitochondria.

## Introduction

Ctenophores, commonly called comb jellies, are a phylum of gelatinous marine animals found in the epipelagic through the abyssopelagic habitats in both planktonic and benthic forms (Pang & Martindale, 2008). Despite the fact that ctenophores are abundant in the water column (Robison, Sherlock & Reisenbichler, 2010) they are difficult to collect and maintain in a laboratory setting (Haddock, 2004; Harbison, 1985b). As a result, we know relatively little about ctenophore biology (Dunn, Leys & Haddock, 2015) and past comparisons to other phyla have confirmed that ctenophores share few qualities with other animals (Harbison, 1985a).

One area of ctenophore “hidden biology” that has come to light is the unique features of ctenophore mitochondrial genomes. The five previously sequenced mitogenomes (*Mnemiopsis leidyi*, *Pleurobrachia bachei*, *Coeloplana loyai*, *Coeloplana yulianicorum* and *Vallicula multiformis*) share a set of characteristics that is unique among metazoans, including rapid mitochondrial evolutionary rates, an absence of mitochondrially-encoded tRNAs, and a lack of mitochondrially-encoded ATP8 and ATP6 (Pett et al., 2011; Kohn et al., 2012; Arafat et al., 2018). While these traits have been explored in past publications, little is known about another common feature to all of these mitogenomes: open reading frames (ORFs) with no known function. Such ORFs are also called unidentified reading frames (URFs).

The presence of URFs in all sequenced ctenophore mitogenomes is striking considering that most metazoan mitochondrial genomes only have the same 13 conserved protein-coding genes (Boore, 1999). Moreover, there are very few examples of metazoans with mitochondrial URFs (Endo et al., 2005; Park, Song & Won, 2011). Importantly, these URFs do not appear to be similar to one another at both the nucleotide and protein level (Arafat et al., 2018). To determine the biological significance of the mitochondrial URFs it is first necessary to determine if they truly encode proteins.

One line of evidence that suggests that some of the ctenophore URFs truly encode proteins is that the *M. leidyi* mitochondrial URFs have high AT frequencies at the third-codon positions (Pett et al., 2011). In addition, all platyctenid (benthic ctenophore) mitochondrial URFs putatively encode transmembrane domains (Arafat et al., 2018). This is significant given that transmembrane domains are a defining feature in characterized mitochondrial metabolic pathway proteins (Becker et al., 2009). While there is no conclusive experimental evidence that ctenophore mitochondrial URFs are protein coding there are several types of computational hypotheses that could strengthen the hypothesis.

Purifying selection on amino acids drives protein-coding loci to have fewer nonsynonymous mutations than synonymous mutations (Graur & Li, 2000). Therefore, evidence of purifying selection in an URF is evidence that it is a translated protein. Using sequence alignments from multiple individuals, one can estimate the nonsynonymous diversity (π*N*) and synonymous diversity (π*S*) of a locus. A ratio of π*N*/π*S* less than one indicates that the locus is under purifying selection (Choi et al., 2016), while a π*N*/π*S* ratio above one indicates that the locus is under balancing selection (Weedall & Conway, 2010; Moncla et al., 2016). However, analyses of π*N*/π*S* have not been performed on published ctenophore mitochondrial URFs since there is only one sequenced individual per species.

Other genic prediction techniques include using trinucleotide frequency over a sliding window (Staden, 1984; Tramontane & Macchiato, 1986; Fickett, 1982) or using a Fourier transform of the nucleotide periodicity (Tiwari et al., 1997; Issac et al., 2002). The afforementioned techniques are useful when the translation table is poorly understood for the target species (Staden, 1984), when the operon-like mitochondrial transcription (Boore, 1999) prevents RNA-seq data from being reliably used to delimit gene boundaries, or when the transcription start site may be immediately after the previous gene’s transcription termination site (Fickett, 1982). These existing trinucleotide-based methods have shortcomings in that they do not account for information from multiple individuals, nor from verified protein-coding ORFs.

**Figure 1:**
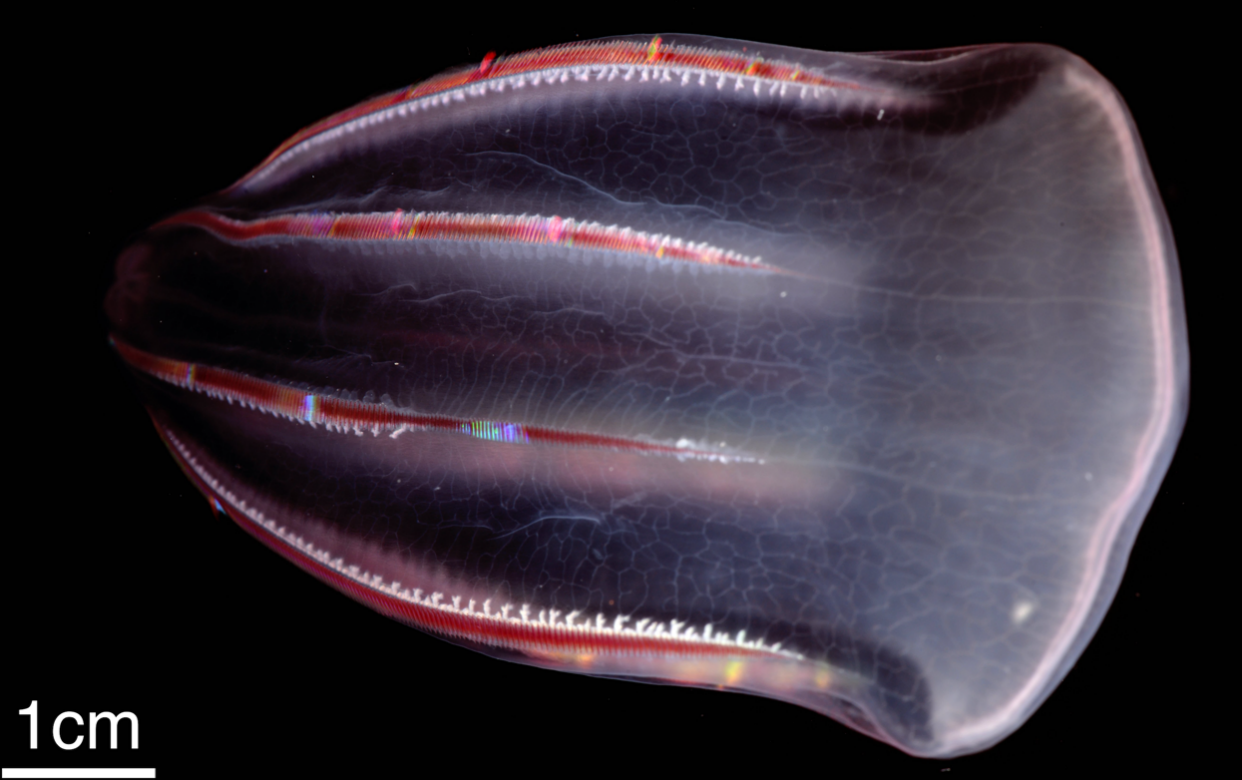
The ctenophore *Beroe forskalii*.

In this study we sequenced and annotated the mitochondrial genomes of three individuals of the ctenophore *Beroe forskalii* (Fig. 1), determined their phylogenetic relationship to sequenced ctenophores, and developed novel algorithms that leverage the multi-individual data to determine if URFs are protein-coding or exist by random chance. In addition to using the measure of π*N*/π*S* to assess if the URFs were under selection, we implemented a novel nucleotide diversity permutation simulation (NDPS) to determine the probability that the URFs arose from negative selection rather than random mutation. To address the limitations of existing trinucleotide genic prediction techniques we developed a novel Bayesian hypothesis test that uses trinucleotide frequencies of known coding and noncoding sequences from multiple individuals to calculate the likelihood that mitochondrial URFs are protein-coding.

## Materials and Methods

### Sample acquisition and sequencing

We collected four *B. forskalii* individuals in the Monterey Bay operating from the Monterey Bay Aquarium Research Institute’s (MBARI) RV *Western Flyer* while blue water diving or using the ROV *Doc Ricketts*. All ctenophore samples were collected under the State of California Department of Fish and Wildlife scientific collecting permit SC-4029 issued to SHDH. See Table S1 for detailed collection information. After allowing the animals’ guts to clear for several hours in on-board aquaria, we froze tissue samples in liquid nitrogen.

We then extracted DNA from *Bf201311* using the Qiagen DNeasy DNA isolation kit (Catalog Number 69504). The sequencing center at the University of Utah Huntsman Cancer Institute High Throughput Genomics Core Facility constructed an Illumina whole-genome shotgun library with an insert size of approximately 250 bp (library 10673X1) and sequenced approximately 124 million 2 × 100 read pairs in December 2013.

We extracted DNA from samples *Bf201606* and *Bf201706* with the Omega Biotek EZNA Mollusc DNA kit (Product Number D3373) and created Illumina whole-genome shotgun libraries with a mean insert size of approximately 150 bp using the NEBNext Ultra II DNA Library Prep kit. We then sequenced these libraries on an Illumina MiSeq and generated approximately 1.74 million 2 × 75 read pairs for *Bf201706*. For sample *Bf201606* we generated Illumina 2 × 150 reads for two libraries: DS117-approximately 122 million read pairs, and DS118-approximately 42 million read pairs. In addition to Illumina sequencing, we constructed five Oxford Nanopore Technologies (ONT) 1D sequencing-by-ligation libraries (SQK-LSK108) from *Bf201606* using 1–1.5 μg DNA as input and two ONT Rapid Sequencing libraries using 200 ng of DNA as input. Each library was sequenced on its own R9.4-chemistry flowcell, and all together the seven flowcells produced 1,772,337 ONT reads with an average length of 4,170 bp.

We also extracted RNA from *B. forskalii* individual *Bf201507* using a Trizol protocol. Two dual-indexed RNA-seq libraries were constructed at the University of California Davis DNA Technologies Core, after which these libraries were sequenced to approximately 175 million 2 × 150 read pairs each on a HiSeq 4000.

Adapters were removed from the Illumina shotgun and RNA-seq libraries using SeqPrep2 version commit 93fccac https://github.com/jeizenga/SeqPrep2 with default parameters and additional option -A GATCGGAAGAGCACACG -B AGATCGGAAGAGCGTCGT. We basecalled the ONT 1D reads using albacore v1.0.3.

### Assembly

We first assembled the *Bf201606* 1D ONT reads with the canu v1.6 assembler using the options genomeSize = 150 m -stopOnReadQuality = false -nanopore-raw (Koren et al., 2017). Then we identified the completely assembled mitochondrial genome contig by using our internal database of ctenophore COI sequences as a blastn query against a database of canu’s contig file output. The contig that contained the ctenophore COI gene appeared to be two full copies of the mitochondrial genome as evidenced by a dotplot (Fig. S3 and Supplemental GitHub/Zenodo Files). We polished this contig by mapping the 1.7 million ONT genomic reads against the assembly with bwa mem and nanopolish https://github.com/jts/nanopolish (Li, 2013). The final consensus sequence was generated by mapping the *Bf201606* Illumina shotgun read pairs against the polished contig with bwa mem, then correcting the remaining misassemblies with several iterations of polishing with pilon v1.22 (Walker et al., 2014) and inspecting the final bam file. We then verified the circularity of the assembly by mapping ONT reads using the -x ont2d option in bwa mem to a fasta file of two concatenated copies of the final assembly.

We used pilon to produce reference-guided assemblies for *Bf201311* and *Bf201706* by using their respective Illumina shotgun reads to correct the *Bf201606* final mitochondrial assembly. As above, we verified the final assemblies of both *Bf201606* and *Bf201706* by visually inspecting a bam file of mapped reads for consistent coverage, and checking for circularity with read pairs that map to opposing ends of the linearized genome.

### Annotation and synteny

After confirming that the assembly was circular and contained no errors we used the MITOS web server to generate an initial annotation of the *Bf201606* mitochondrial genome (Bernt et al., 2013b). Then we used emboss v6.6.0 to find ORFs in sample *Bf201311* (Rice, Longden & Bleasby, 2000). ORFs were annotated using results from the blastn, tblastx, and blastx (v2.2.31+) queries against NCBI databases (Altschul et al., 1997). We then aligned all three individuals’ mitochondrial genomes in Geneious v9 using the Geneious aligner. The final ORF boundaries for each *B. forskalii* individual were defined using the largest conserved ORF shared between all three individuals. We calculated π and the average between-sample mismatch percent of the complete mitochondrial genome alignment using the equation from Nei & Li (1979), implemented in cuttlery calculate-pi.

To annotate the tRNAs, we used tRNAscan-SE v2.0 and ARWEN v1.2 (Lowe & Eddy, 1997; Laslett & Canback, 2008). To determine the start and stop points of the ribosomal RNAs (rRNAs), we created covariance models of the *M. leidyi* 12S and 16S ribosomal RNAs from (Pett et al., 2011) using infernal v1.1 (Nawrocki & Eddy, 2013). In addition, we mapped RNA-seq reads to the mitochondrial genome using bwa mem to look for coverage minima to help determine the start and stop points of the rRNAs (Li, 2013).

To search for ATP6 and ATP8 in the *B. forskalii* nuclear genome, we assembled a transcriptome and searched with BLAST v2.2.31+. We assembled the 175 million *Bf201706* RNA seq reads using Trinity v2.1.1 with the option -SS_lib_type FR for read directionality. We then searched for ATP6 using blastn and tblastx with the *M. leidyi* ATP6 sequence, ML33722a, and the *P. bachei* ATP6 sequence (sb*|*11606431*|*).

Many species have nonstandard codons in their mitochondrial amino acid genetic code Barrell, Bankier & Drouin (1979); Knight, Freeland & Landweber (2001). Nonstandard codons can be inferred by comparing conserved codons in conserved genes shared across phyla. Ctenophores are thought to use the Mold, Protozoan, and Coelenterate mitochondrial translation table (Pett & Lavrov, 2015). We used the software FACIL to look for genetic code deviations from the Mold, Protozoan, and Coelenterate mitochondrial translation table in all three *B. forskalii* mitochondrial genomes (Dutilh et al., 2011).

We implemented pauvre redwood to visualize sequencing reads supporting the assembly, and implemented cuttlery codonplot to plot the codon usage distributions for each mitochondrial ORF. To quantify the syntenic differences between the three ctenophore species, we used CREx (Bernt et al., 2007).

### Phylogenetics

To make a phylogeny of ctenophores plus outgroups we followed the protocol found in Arafat et al. (2018). Ctenophores and outgroups were collected from NCBI (Lang et al., 2002; Kohn et al., 2012; Fallon et al., 2018; Arafat et al., 2018; Naylor & Brown, 1998; Qureshi & Jacobs, 1993; Paquin & Lang, 1996; Forget et al., 2002; Ogoh & Ohmiya, 2004; Seif et al., 2005; Lavrov, Wang & Kelly, 2008; Wang & Lavrov, 2008; Lavrov et al., 2005; Akasaki et al., 2006; Dellaporta et al., 2006; Shao et al., 2006; Bourlat et al., 2006; Signorovitch, Buss & Dellaporta, 2007; Lukić-Bilela et al., 2008; Tambor, Ribichich & Gomes, 2008; Matsui et al., 2009; Erpenbeck et al., 2009; Gazave et al., 2010; Pett et al., 2011; Kayal et al., 2012; Zou et al., 2012; Park et al., 2012; Pan et al., 2014, Del Cerro et al., 2016; Jourda et al., 2015; Haen, Pett & Lavrov, 2014; Li, Sung & Ho, 2016; Chen et al., 2016; Poliseno et al., 2017; Wang & Sun, 2017; Galaska et al., 2019), then an amino acid matrix was generated using MAFFT alignments of COX1, COX2, COX3, CYTB, ND1, ND3 and ND5. MAFFT parameters were: v7.309 with the FFT-NS-i x1000 algorithm with a 200PAM *k* = 2 scoring matrix, 1.53 gap open penalty, and 0.123 offset value (Katoh et al., 2002). Low-information columns were removed with Guidance2 (Sela et al., 2015). We also generated an amino acid matrix with no columns removed. In addition to these two amino acid matrices, we similarly generated one amino acid matrix, without Guidance2 filtering, using only ctenophores plus two outgroups, and genes COX1, COX2, COX3, CYTB, ND1, ND2, ND3, ND4, ND4L, ND5 and ND6. Guidance2 was not used to ensure that we did not remove columns important to resolving inter-ctenophore relationships. Note, we used the updated *P. bachei* annotation from Arafat et al., 2018, and that hexactinellids have AGR codons translated as serine rather than arginine (Arafat et al., 2018; Haen et al., 2007). A detailed sample list is available in Table S7.

For each of these three matrices we created maximum likelihood trees using RAxML and Bayesian consensus trees using Phylobayes. For RAxML we used v8.2.4 using rapid bootstrapping, one partition for all sites, and the PROTCATWAG model with seeds -p 12345 -x 12345 (Stamatakis, 2014). For Bayesian consensus trees we used Phylobayes v4.1c and ran three chains until the maximum difference was less than 0.1 and the minimum effective size was greater than 300, indicating that all three chains had converged appropriately (Lartillot, Lepage & Blanquart, 2009). See Tables S8–S10 for details about the Phylobayes runs.

### Fourier transform analysis

To compare the predictions of the Bayesian protein coding likelihood test to an established method, we used the FTG webserver and the FTG-WINDOW program with a step size of 20 and window size of 300 to look at the Fourier Transform nucleotide spectra of the three *B. forskalii* mitochondrial genomes (Issac et al., 2002; Tiwari et al., 1997). The Fourier Transform nucleotide spectrum analysis looks at an increased periodicity in trinucleotide usage over a sliding window in the DNA sequence. Areas with a regular periodicity have a higher score of being protein coding. We expected to see peaks centered around the known coding ORFs, and peaks in the URF1 and URF2 sequences if truly protein coding.

### Protein structure and function prediction

We generated the peptide sequences using the mold and protozoan mitochondrial translation table for all of the *B. forskalii* and previously sequenced ctenophore URFs. Transmembrane predictions for each protein were made with the TMHMM server, and with MEMSAT-SVM on the PSIPRED server (McGuffin, Bryson & Jones, 2000). We predicted *Bf201706*’s URF1 and URF2 3D protein structures using the I-TASSER web server (Roy, Kucukural & Zhang, 2010) and DMPfold 1.0 on the PSIPRED server (McGuffin, Bryson & Jones, 2000). Use used FFPred 3 to predict the function of the URF proteins (McGuffin, Bryson & Jones, 2000). I-TASSER and FFPred 3 allowed us to look for putative functions for the URFs in the absence of primary structure orthologs using blastp. We also used FFPred and MEMSAT-SVM to analyze the TM domains and functions of the URFs from *P. bachei*, *M. leidyi*, *C. yulianicorum*, *C. loyai*, and *V. multiformis*.

To look for orthologs of *B. forskalii* URF1 and URF2 we used blastn and blastp to search the nt and nr databases, respectively (Altschul et al., 1997). To look at the similarity between URFs we used Clustal Omega to form a multiple sequence alignment (Sievers et al., 2011).

### Bayesian protein coding likelihood test

Bayesian hypothesis tests are a method to determine whether a given dataset better matches one hypothesis or another (Ortega & Navarrete, 2017). Given that there we could not find software to help determine if mitochondrial URFs were protein coding or not, we developed a Bayesian hypothesis test. This test classifies whether the codon frequency spectrum of an ORF best matches the codon frequency spectrum of known protein-coding sequences or the 3-mer frequency spectrum of known non-coding sequences. This methodology is based on the fact that 3-mer frequency varies between coding and non-coding sequences (Hinds & Blake, 1985; Staden, 1984).

Given that there are 62 amino-acid encoding codons to model, we required a distribution with 62 parameters that could be applied to a Bayesian framework. Dirichlet distributions are comprised of probability vectors and are conducive to Bayesian computation, as the distribution is the prior for the multinomial distribution (Kotz, Balakrishnan & Johnson, 2004). We modeled an ORF’s codon frequency spectrum as a multinomial random variable that can be drawn from either a coding or non-coding distribution. Each of these distributions was parameterized by a probability vector from the posterior Dirichlet distribution given the known coding and non-coding sequences. The test statistic then consisted of the Bayes factor that the ORF was drawn from the coding distribution (See a more detailed exposition in the Supplemental Methods). The Bayes factor allowed us to assess if factors such as sequence length limited the amount of evidence that the hypothesis had to classify that specific locus (Ortega & Navarrete, 2017).

We empirically evaluated the sensitivity and specificity of this test using the known coding and non-coding sequences and performed 1,000,001 leave-one-out cross-validation (LOOCV) trials. In each trial we randomly choose a sequence with a known coding status from a random individual. In non-coding sequence we also randomly choose a reading frame. We then computed the Bayes factor for this sequence with the posterior distributions conditioned on only the remaining sequences. To ensure that each locus is independent, we only used one individual’s copy of each sequence to condition the posterior distributions in each trial.

The coding ORFs that condition the posterior are the genes identified in the annotation (COX1, COX2, COX3, CYTB, ND1 through ND6). The non-coding sequences are all of the non-rRNA intergenic sequences of at least 50 basepairs—excluding the novel ORFs themselves. To avoid strand biases, we only selected non-coding sequences on the same strand as the coding genes. We opted to not use the opposite strand of coding sequences since their sequences are affected by the constraints on the coding sequence. To prevent the software for classifying sequences solely on start or stop codons, we removed internal and flanking stop codons from non-coding sequences, stop codons from the end of true ORFs, and start codons from the beginning of both non-coding sequences and ORFs. Additional details on the test are included in the Supplemental Material. This program is implemented in cuttlery dirichlet. We also validated this method on gene alignments from a variety of organisms from diverse groups across the tree of life including the five individuals of the alga *Chlamydomonas reinhardtii* (Smith & Lee, 2008), nine individuals of the crustacean *Daphnia magna*, thirteen individuals of the fly *Drosophila melanogaster* (Wolff et al., 2016), five individuals of the vertebrate *Homo sapiens* (Yang et al., 2009; Rani et al., 2010; Guillet et al., 2010, Van De Loosdrecht et al., 2018), and four individuals of the urchin *Strongylocentrotus intermedius* (Kober & Bernardi, 2013). See Table 1 for a list of accession numbers used and the Zotero repository for alignments.

**Table 1:** NDPS results for *B. forskalii* ORFs and URFs. These π and π*N*/π*S* values were measured using biopython’s cal dn ds function, and π*N*/π*S* was measured using the NG method (Nei & Gojobori, 1986). The Monte-Carlo *p*-value is estimated from the Nucleotide Diversity Permutation Simulation and measures the probability that this locus is evolving neutrally. This Monte-Carlo *p*-value was calculated by counting the number of simulated π*N*/π*S* less than the observed π*N*/π*S* that also had π*S* values greater than zero, and dividing by the number of simulations for which π*S* was greater than zero. All loci, including the URFs, appear to be evolving under negative selective pressure. The ND4L locus’ high MC *p*-value is likely an artifact of the short sequence length.

Sequence	π	π*N*/π*S*	MC *p*-value
COX1	0.0094	0.0124	0.0000
COX2	0.0032	0.0000	0.0000
COX3	0.0027	0.0650	0.0000
CYTB	0.0085	0.0120	0.0000
ND1	0.0050	0.0122	0.0000
ND2	0.0075	0.0267	0.0000
ND3	0.0048	0.0285	0.0000
ND4	0.0058	0.0353	0.0000
ND4L	0.0040	0.4329	0.4191
ND5	0.0044	0.0710	0.0000
ND6	0.0066	0.0410	0.0000
URF1	0.0055	0.3497	0.0120
URF2	0.0214	0.2477	0.0000

### Nucleotide diversity permutation simulation

We did not have outgroups to perform a McDonald–Kreitman test (McDonald & Kreitman, 1991), and therefore we were limited to estimating π*N*/π*S* to detect negative selection (Choi et al., 2016; Weedall & Conway, 2010; Moncla et al., 2016). In addition to measuring negative selection with π*N*/π*S* we wanted evidence that a π*N*/π*S* value less than one was the result of negative selection, and not a false positive. To gather such evidence we devised a test to generate Monte Carlo *p*-values that compare the observed π*N*/π*S* value to a null distribution. In this case the null distribution was a collection of π*N*/π*S* values estimated from simulated sequences generated through a neutral evolution process. For ease of computation of a large number of sequences using the cal_dn_ds method in biopython (Cock et al., 2009), and because the mutations generated were random, we selected the Nei & Gojobori (1986) method of calculating dN, dS, and subsequently π*N*/π*S*. For a more detailed exposition of the nucleotide diversity permutation simulation (NDPS), see the Supplemental Methods.

We also tested this program on the non-*Beroe* species listed in the Bayesian protein coding likelihood test methods section. See Table 1 for a list of accession numbers used.

### Software

We implemented and made freely available two python software packages to complete the analyses in this manuscript. The pauvre software can be found at https://github.com/conchoecia/pauvre, and it contains a program to visualize and verify mitochondrial assemblies using long reads (pauvre redwood), and a tool to visualize synteny between mitochondrial genomes (pauvre synplot) (De Coster et al., 2018; Bentley & Ottmann, 1979).

We also developed the software package cuttlery to implement the NDPS (cuttlery piNpiSsim), to conduct the trinucleotide protein-coding test (cuttlery dirichlet), to plot the clustering of π*N* > 0 and π*S* > 0 sites along a gene similar to Carbone et al. (2006), and to calculate nucleotide diversity (cuttlery calculate-pi). The program cuttlery calculate-pi was validated using DNAsp (Rozas et al., 2003). This software is available at https://github.com/conchoecia/cuttlery.

A script written with Snakemake (Köster & Rahmann, 2012) that reproduces many of the plots found in this manuscript is available in the Supplemental GitHub/Zenodo Data.

## Results

### Assembly

**Figure 2:**
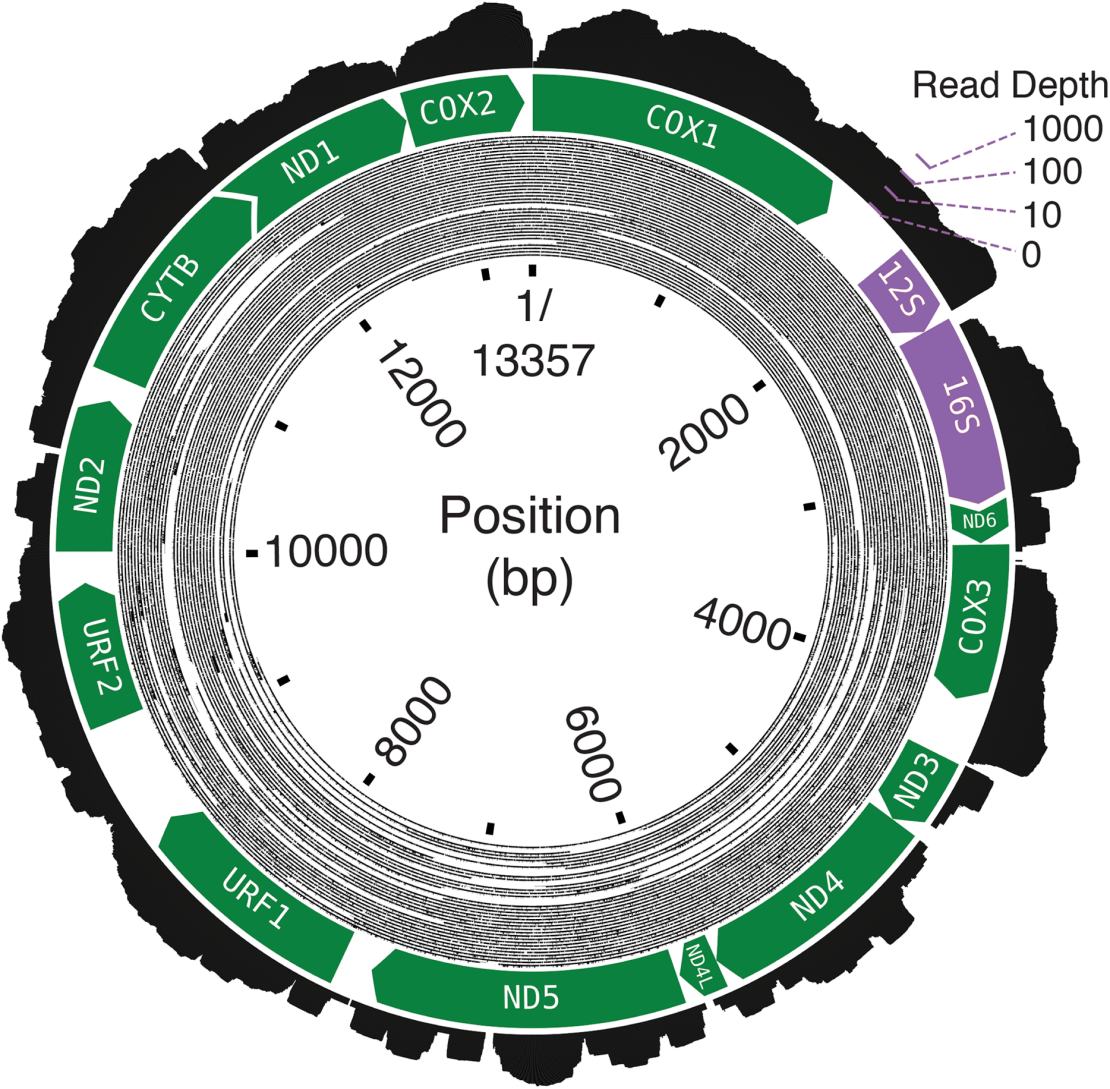
The *B. forskalii* mitogenome. Each black concentric circle of the inner layer is one Oxford Nanopore read, organized from the longest reads on the outside of the track to the shortest on the inside. The annotation shows the direction and length of the predicted coding sequences (green) and the ribosomal RNAs (purple). Overlapping coding sequences are shown with an overlapping chevron on the 5′ end of the downstream gene. The outermost layer is a histogram of RNA-seq log-transformed read coverage at that position.

The final assembly lengths for the Bf201311, Bf201606, and Bf201706 mitochondrial genomes were 13,357, 13,339 and 13,338 basepairs. Bf201311’s mean mapping coverage was over 6,000× with 2 × 100PE trimmed reads, and the mean mapping coverages of both Bf201606 and Bf201706 mitochondrial genomes with 2 × 75PE trimmed reads were approximately 70×. Oxford Nanopore 1D reads confirm its circularity (Fig. 2). The mean per-basepair divergence between individuals was 2.2%. The mean GC content was 17.3%.

The size discrepancy between these three sequences is due to small insertions and deletions of one to 16 basepairs (Table S2). There were no indels in any of the three individuals’ canonical mitochondrial genes, nor in URF2 and URF1. Whole-mitogenome alignments can be found in the Supplemental GitHub/Zenodo Files.

### Annotation and synteny

The *B. forskalii* mitochondrial genome contains the same two ribosomal RNAs (12S and 16s), and the same 11 protein-coding genes as *M. leidyi*: COX1, COX2, COX3, CYTB, ND1, ND2, ND3, ND4, ND4L, ND5 and ND6. The *B. forskalii* mitochondrial genome lacks the ATP6 and ATP8 genes.

In addition to the canonical mitochondrial coding sequences above, there are two large URFs in the *B. forskalii* mitochondrial genomes that are conserved among all three individuals. We refer to the first large ORF that begins after ND5 on the coding strand as URF1. This sequence is 1,065 bp and encodes a putative 384 aa protein. We refer to the second large conserved URF after URF1 as URF2. URF2 is 669 bp and encodes a putative 222 aa protein. These ORFs have no matches to NCBI databases with BLASTn, BLASTx, PSI-BLAST and tBLASTn. Also, neither URF appears to have any homology with published ctenophore genomes.

Our structural models of *M. leidyi* 12S and 16S ribosomal RNAs had significant matches to the 3′ ends of both the *B. forskalii* 12S and 16S sequences. We verified the start and stop sites using Illumina RNA-seq data. The structural orthology and clear read delimitation information allowed us to annotate both the 5′ and 3′ ends of the 12S and 16S ribosomal RNAs.

A tRNA search using tRNAscan-SE with infernal did not identify any tRNAs in any of the three *B. forskalii* mitochondrial genomes. ARWEN did not identify any tRNAs that were conserved between the three individuals. ARWEN detected one TV-loop mtRNA-Phe(aaa) in Bf201706 in the middle of the 16S sequence, one TV-loop mtRNA-Ser(act) in the middle of the COX2 coding sequence in 201311, and no tRNAs in Bf201606.

**Figure 3:**
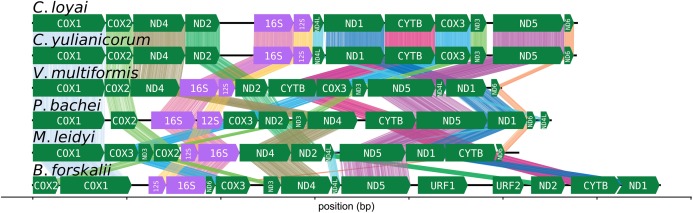
Ctenophore mitochondrial synteny map. A synteny map of the ctenophore mitochondrial genomes. The opacity of the “brush stroke”-like bars connecting the same gene between two species increases with positional amino acid similarity using the BLOSUM62 matrix. Exact matches for ribosomal RNAs are opaque lines, while mismatches and gaps are not displayed. This plot was generated using the program pauvre synteny.

The CREx heuristic revealed that *B. forskalii* shares more common intervals with *M. leidyi* (16 common intervals) than with *P. bachei* (eight common intervals), *V. multiformis* (10 common intervals), or *Coeloplana* spp. (eight common intervals). There is considerable gene order shuffling between the three species (Fig. 3).

The FACIL analysis did not reveal any deviations in the genetic code from the Mold, Protozoan, and Coelenterate translation table (See Table S15; Fig. S12).

### Phylogenetics

**Figure 4:**
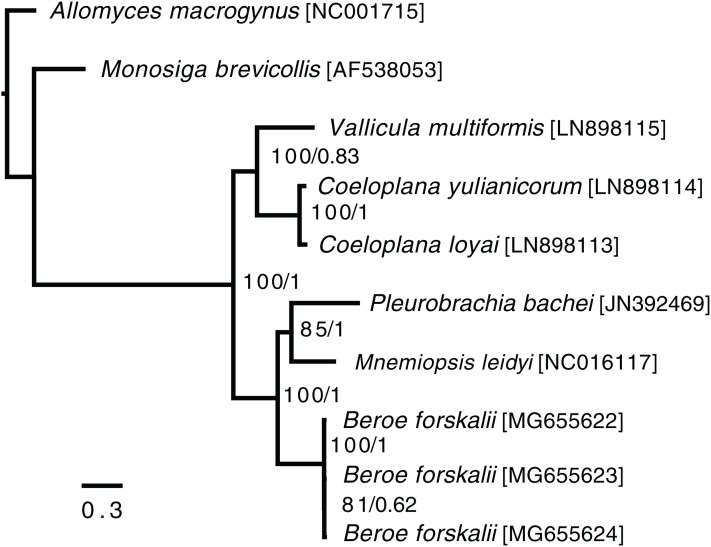
Ctenophore mitochondrial phylogeny. This phylogeny contains the loci COX1, COX2, COX3, CYTB, ND1, ND2, ND3, ND4, ND4L, ND5 and ND6 independently aligned with MAFFT (Katoh et al., 2002) then concatenated together. No sites were removed from the amino acid matrix. Two phylogenies were created (1) using RAxML with rapid bootstrapping and (2) using Phylobayes with the CAT + GTR + Γ model, three chains, and convergence until the max difference between chains was less than 0.1. Both trees reconstructed the same topology. The branch lengths and scale shown are from the RAxML tree. The RAxML bootstrap values/Phylobayes posterior probabilities of each node are shown within the ctenophore clade.

In the ctenophore/two-outgroup amino acid matrix, the monophyly of ctenophores is well-supported (bootstrap value of 100, posterior probability of one) (Fig. 4). The relationship of platyctenid ctenophores as a monophyletic clade that is, sister to the rest of the ctenophores was supported in the RAxML trees but not the Phylobayes trees.

Trees that were constructed with only a limited set of mitochondrial proteins (COX1, COX2, COX3, CYTB, ND1, ND3 and ND5) and used Phylobayes CAT + GTR + Γ model failed to reconstruct platyctenid ctenophores as monophyletic, with or without removing sites using Guidance2. Trees constructed with the same set of genes using the PROTCATWAG model in RAxML reconstructed platyctenes as a monophyletic group. The hexactinellid sponges, in all trees in which they were included (Figs. S7–S10) did not form a monophyletic group with the rest of the sponges. Early-divergence relationships, such as the evolutionary branching order of Sponges, Ctenophores, Choanoflagellates, and Cnidarians, had low bootstrap support and low posterior probability values.

### Ctenophore mitochondrial URF structure and function prediction

*Beroe forskalii* URF1 and URF2 had no blast hits in the nr or nt databases, with no cutoff. A multiple sequence alignment between all of the URFs in all ctenophore species showed that while most of the *Coeloplana* sequences were generally similar to one another (28–83.3% identity), the between-genera alignments had generally fewer than 30% identical amino acids (Table S4). This is below a commonly-used threshold for orthology (Pearson, 2013). Protein alignments with high percent identity were exclusively from short peptide sequences. However, shorter peptide sequences are more likely to have high amino acid identity by chance (Pearson, 2013). Overall, these results indicate that *B. forskalii* URF1 and URF2 have no known orthologs, and are not similar to any other published ctenophore URFs.

We predicted the protein structure of URF2 using I-TASSER, and the top five predicted models are transmembrane-protein-like and are composed of antiparallel alpha helices. Models 1,2,3 and 5 all contained six alpha helices and model four contained eight alpha helices. The top five structural hits were transmembrane transport proteins, including one protein with an unidentified transport substrate (PDB 3WDO), a peptide transport protein (4W6V), an iron transport protein (5AYM), a nitrate transporter (5A2N), and a proton:xylose symporter (4GBY). TMHMM predicted 6 transmembrane domains with an extracellular N-terminus, while MEMSAT-SVM predicted seven transmembrane domains with a cytoplasmic N-terminus. FFPred’s top predicted biological process for URF2 was GO:0055085, transmembrane transport (Prob = 0.774), and the top molecular function prediction was GO:0022857, transmembrane transporter activity (Prob = 0.875) (Table S3).

The predicted URF1 structure was also similar to known transmembrane proteins. The top five structural predictions were composed of between 8 and 11 antiparallel transmembrane domains. TMHMM predicted eight transmembrane domains with an intracellular N-terminus while MEMSAT-SVM predicted nine transmembrane domains with an extracellular N-terminus. The transmembrane domains boundaries were largely the same, except for the last two domains predicted by both tools. The highest-scoring structural hit had a high structural similarity TM-score of 0.941, and is an iron transport protein (PDB 5AYM). PDB 5AYM is the same protein as the third-best hit for the URF2 structural hit above. Like the URF2 structural hits, all of the URF1 structural hits are to transmembrane transport proteins. The top biological process predicted by FFPred was GO:0098655, cation transmembrane transport (Prob = 0.879), and the top molecular function prediction was GO:0022857, transmembrane transporter activity (Prob = 0.951) (Table S3).

Overall, the URFs from the other ctenophore species had similar GO annotations to *B. forskalii* URF1 and URF2. Every URF from *P. bachei*, *M. leidyi*, *C. yulianicorum*, and *C. loyai* had the term *transport* in at least one of the top three Biological Process GO terms predicted by FFPred. All of the URFs with at least two predicted transmembrane domains had Molecular Function GO terms with high probabilities related to transmembrane transporter activity. Other common molecular function GO terms were GO:0003824 catalytic activity, GO:0005125 cytokine activity, GO:0008270 zinc ion binding, and GO:0001882 nucleoside binding (Table S3). URF1 from *V. multiformis* was too short to analyze using PSIPRED.

### Observed π*N*/π*S*

All of the canonical genes (ND1-ND5, ND4L, COX1-COX3, CYTB), with exception of ND4L, had π*N*/π*S* values between zero (COX2, no non-synonymous sites) and 0.652 (ND4L). The mean π*N*/π*S* of the canonical genes is 0.087. ND4L is the shortest of the sequences observed in this analysis at 189 bp and only three mutation sites. The π*N*/π*S* of URF1 and URF2 are 0.350 and 0.248, respectively. These results suggest that the URF1 and URF2 ORFs are under less selective pressure than the canonical ND1-6/COX1-3/CYTB genes. The π*N*/π*S* values calculated for the non-*Beroe* test datasets were less than one for invertebrate species, similar to results found in similar studies such as Bazin, Glémin & Galtier (2006) and Rand & Kann (1998).

We also looked at the distribution of nonsynonymous mutation sites in the protein structural domains of *B. forskalii* URF1 and URF2 (See Fig. S13; Tables S11–S14). In mitochondrial transmembrane transport proteins each peptide is either transmembrane (TM), exposed to the mitochondrial matrix (MM), or is exposed to the intermembrane space (IM) between the mitochondrial membrane and the outer membrane. We found that both URF1 and URF2 had elevated mutation rates in MM-exposed peptides (Table S13), despite both proteins having fewer MM sites than the IM-exposed peptides and TM peptides (Table S12). Overall, *B. forskalii* URF2 had more mutation sites, both synonymous and nonsynonymous, than *B. forskalii* URF1 (Table S11).

### Nucleotide diversity permutation simulation

The Monte–Carlo *p*-value for all genes in the observed vs absence-of-selection simulated π*N*/π*S* experiment was *p* < 0.001, except ND4L and URF1 (Table. 1). ND4L had a Monte–Carlo *p*-value of *p* = 0.4191 and URF1 had a Monte–Carlo *p*-value of *p* = 0.0120. The π*N*/π*S* value of ND4L (0.433) was an outlier compared to the mean canonical mitochondrial genes’ π*N*/π*S* values (Fig. S5), but this is likely due to only three polymorphic sites in a short sequence of 186 basepairs (Table S11). The π*N*/π*S* values of URF1 and URF2 were also outliers relative to the mean canonical gene π*N*/π*S*, at 0.350 and 0.248 (Table 1). All of the canonical mitochondrial genes have a lower observed π*N*/π*S* than the range of π*N*/π*S* values predicted by the nucleotide diversity mutation simulation (*p* = 0) with the exception of ND4L (*p* = 0.419) and URF1 (*p* = 0.012) (Fig. S5; Table 1).

The results of this simulation for non-*Beroe* species were generally that the Monte Carlo *p*-value accurately predicted that a sequence’s π*N*/π*S* were due to negative selection rather than neutral mutations (Table 1; Table S6; Fig. S5). The outliers were mostly in the human mitochondrial data, which do not have as much negative selective pressure on mitochondrial loci as invertebrates (Meiklejohn, Montooth & Rand, 2007), and overall have less nucleotide diversity than invertebrates (Bazin, Glémin & Galtier, 2006).

### Bayesian protein coding likelihood test

**Figure 5:**
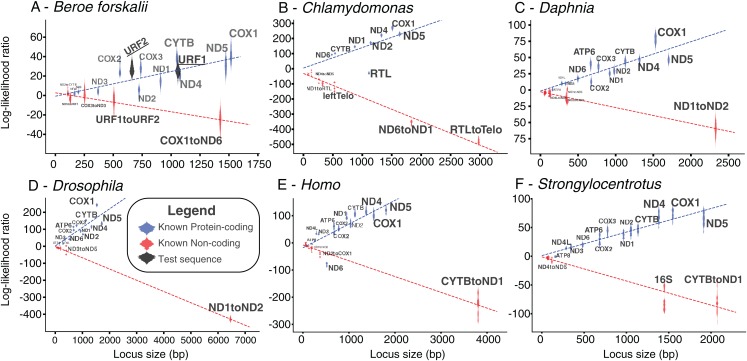
Bayesian likelihood of ORFs being coding or noncoding. These plots show the locus length versus the log-likelihood ratio distributions of the LOOCV trials for each noncoding, coding sequence, or test sequence (*B. forskalii* URF1 and URF2). (A) *Beroe forskalii*, (B) *Chlamydomonas*, (C) *Daphnia*, (D) *Drosophila*, (E) *Homo*, (F) *Strongylocentrotus*. Dotted lines are linear fits to the log-likelihood values for each simulation. Log-likelihood ratios less than zero mean that the sequence’s trinucleotide frequency was more similar to the trinucleotide frequency of noncoding sequence than that of known protein-coding sequence. Similarly, values greater than zero indicate a better match to known protein-coding sequences. For all species we tested, with the exception of ND6 in human and RTL in Chlamydomonas, we found that the novel Bayesian likelihood test for protein coding likelihood presented in this paper unambiguously can differentiate between coding and noncoding sequences for loci longer than 500 bp. The alignments used in these analyses are the same as those used in Table S5; Fig. S5.

The log-likelihood ratio distributions for URF1 and URF2 more closely match those found in other known coding sequences even when the effects of sequence length were considered (Fig. 5). The power of both analyses were approximately 0.97. The results of this analyses performed on other species also show that most loci longer than 500 bp are clearly distinguishable as coding or non-coding (Fig. 5).

### Codon usage frequency and FTG analysis

The codon usage frequencies of genes in the *B. forskalii* mitochondrial genome are skewed toward AT-rich codons (Fig. S4). The Fourier Transform FTG-WINDOW analysis shows trinucleotide periodicity above the signal-to-noise threshold that corresponds to protein-coding sequence between 0–1,000 bp (COX1), around 6,500 bp (ND5), around 7,800 bp (URF1) and around 9,300 bp (URF2).

## Discussion

### Three *B. forskalii* mitogenomes show high between-individual haplotype variability

The genus *Beroe* represents one of the distinct morphological and taxonomic lineages of comb jellies, and complements the “cydippid” *P. bachei* and lobate *M. leidyi* species whose mitochondrial genomes have been previously studied. Species of *Beroe* can be difficult to distinguish, but *B. forskalii*, which we study here, is morphologically distinct and readily identified. The four specimens collected were from the same waters off the coast of California (See Supplemental Methods 1). Two specimens were from the same geographic location, with one collected at the surface and one collected from 400 m. The third specimen was taken at the surface 70 km to the south, and the fourth specimen was taken at the surface 50 km to the east. Based on the distribution of *B. forskalii* along the eastern Pacific, these three specimens would be expected to belong to the same population, and any molecular differences are intraspecific and not likely to be indicative of biogeographic boundaries.

The COX1 gene, which is widely used for species- and population-level genetic studies, has been difficult to obtain for many ctenophore species due to high intraspecific nucleotide diversity. Standard primers, such as those from Folmer et al. (1994), fail to amplify COX1 sequences from many species. Our current data set helps clarify the diversity of mitochondrial genes from additional ctenophore lineages.

### The *B. forskalii* mitogenome contains the same subset of genes as other sequenced ctenophore

One surprising finding from the first ctenophore mitochondrial genome annotations was that ctenophores lack two or more genes that are conserved in most other metazoan phyla (Kohn et al., 2012; Pett et al., 2011; Arafat et al., 2018). In our dataset, the *B. forskalii* mitochondrial genome is missing the same protein-coding sequences as the other five ctenophore species’ mitogenomes: ATP6 and ATP8. In both *M. leidyi* and *P. bachei*, mitochondrial ATP6 is present in the nuclear genome, although ATP8 is not. There is no publicly available data for platyctenid ctenophores to determine if they similarly have ATP6 and ATP8 in the nuclear genome. We did not have a complete *B. forskalii* nuclear genome to detect the presence of ATP6 or ATP8. However, we were able to detect a *B. forskalii* ATP6 in the transcriptome.

Given that mitochondria are transcribed as operons (Boore, 1999), the uncertainty of whether ctenophores use mitochondrial mRNA 3’ polyadenylation (Chang & Tong, 2012), and the tendency for mitochondrial transcripts to not match gene boundaries (Gao et al., 2016), poly-A RNA-seq libraries are not a reliable way to determine ctenophore mitochondrial transcript start and stop sites. *B. forskalii* poly-A RNA-seq reads mapped to the mitochondrial genome did not reliably help determine gene start and stop positions. A future study of ctenophore RNA using direct RNA sequencing may reveal trends in ctenophore mitochondrial transcription and post-transcriptional modification.

All sequenced ctenophore mitochondrial genomes lack tRNAs, and there is evidence that they are genuinely missing given the lack of nuclear-encoded mitochondrial aminoacyl-tRNA synthetases (Pett & Lavrov, 2015). Our results using standard tRNA identification software did not have any hits conserved between individuals, and each hit was in the middle of another annotated feature (16S in Bf201706 and COX2 in Bf201311). The fact that these hits appear in the middle of other features is strong evidence that they are not true tRNAs. This, however, does not preclude the existence of yet-unidentified mitochondrial tRNAs (mtRNAs) and future studies should use specialized tRNA sequencing methods to map tRNAs to a reference (Cozen et al., 2015).

Ribosomal RNAs are similarly difficult to annotate accurately. In the case of ctenophore mitochondrial genomes, the highly derived ribosomal RNAs often are not recognized by publicly available covariance models found on Rfam and used by infernal. In the case of Pett et al. (2011), the authors used a locally-crafted covariance model to identify the 16S and 12S genes in *M. leidyi*. However, the details of the model were not published and are no longer available from the authors. As a result, we crafted covariance models of the single published *M. leidyi* 16S and 12S nucleotide sequences. These covariance models matched several domains of what MITOS predicted for the 16S ribosomal sequence, and a region upstream in the *B. forskalii* mitochondrial genome matching the 12S covariance model. The 12S gene shares structural similarity with the 12S covariance model in the 3′ region of the gene, although there was little similarity with the 5′ region. This suggests that either the Pett et al. (2011) 12S structural model was incorrect, or that the 12S rRNA structures are not conserved between ctenophore species. Similarly, the published *M. leidyi* 16S rRNA structure lacks information on approximately the first 500 basepairs. Due to the high divergence of ctenophore ribosomal RNAs from the rest of the tree of life, experiments that help determine RNA structure, like SHAPE-seq (Loughrey et al., 2014), would be beneficial to future studies of ctenophore mitochondrial and nuclear ribosomal RNAs.

### Ctenophore mitochondrial rearrangements are common between sequenced genera

The number of common intervals is a metric of how closely related are two mitochondrial genome gene orders (Bernt et al., 2007). While we are not able to determine the exact rearrangement pathway leading to the different gene orders between *B. forskalii* and other ctenophore species, it is clear that large-scale mitochondrial rearrangements occurred during the diversification of ctenophores, similar to the findings of Arafat et al. (2018). One feature to note is that transcription appears to be unidirectional in all six ctenophore species (Fig. 3).

### Beroid ctenophores are sister to a clade containing Pleurobrachia and Mnemiopsis

In the ctenophore/two-outgroup tree *B. forskalii* is sister to a clade of *M. leidyi* and *P. bachei*. This contrasts with a previous transcriptome-based phylogeny that found that *P. bachei* is sister to a clade containing *M. leidyi* and *B. forskalii* (Simion et al., 2017). Further studies on ctenophore phylogenies using an expanded set of species may resolve the discordant nodes, or may raise questions concerning mitochondrial introgression after the platyctenid split.

The Phylobayes results, despite using the same loci and similar outgroups to Arafat et al., 2018, found that platyctenid ctenophores were not a monophyletic clade that is, sister to the rest of the ctenophores. Our RAxML results, however, reconstructed the same topology as found in Arafat et al. (2018). This discrepancy may be due to differences in alignments caused by using different outgroups than Arafat et al. (2018) despite us using the same phylogenetic protocol and loci.

### *Beroe forskalii* URFs are more similar to coding sequences than noncoding

We attempted to use several methods to determine if the *B. forskalii* URFs are protein-coding. First, we checked that the codon usage of the URFs was similar to the codon usage of the canonical ORFs (Fig. S4). URF1 and URF2 generally had similar trinucleotide frequencies to the canonical genes (ND1-6, CYTB, COX1-3), although this information alone was was not sufficient to discern whether URFs were bona fide protein-coding genes.

The results of the Bayesian hypothesis test suggested that the URF1 and URF2 codon usage profile is more similar to the codon usage profile of the canonical coding sequences than to the trinucleotide frequency of the known non-coding sequences (Fig. 5). Overall, the Bayesian hypothesis test’s ability to classify a sequence as noncoding or coding was proportional to the length of the locus in question (Fig. 5). Moreover, the test had a high positive predictive ability for true protein coding genes using mitochondrial datasets for well-studied species (Fig. 5). The Fourier Transform analysis of the *B. forskalii* mitogenomes also indicate URF1 and URF2 are likely protein coding regions (Fig. S6).

### *Beroe forskalii* URFs appear to be under purifying selection

We looked for signatures of selection on the putative amino acid sequences of URF1 and URF2 directly by estimating π*N*/π*S*, and observed that the π*N*/π*S* of the canonical mitochondrial genes and the URF2 and URF1 putative genes were all less than one (Table 1; Fig. S5). These results suggest that the *B. forskalii* URFs are under negative selection. However, the URF1 and URF2 π*N*/π*S* values were higher than the π*N*/π*S* values of the ND1-6, CYTB and COX1-3 genes. This discrepancy raised the question of whether the elevated π*N*/π*S* values in URF1 and URF2 are the result of lower negative selective pressure relative to ND1-6, CYTB and COX1-3 or if URF1 and URF2 evolved in the absence of selective pressure. To clarify this question we devised a simulation in which we mutated the ORFs randomly while preserving the phylogenetic relationship and nucleotide diversity observed between the ORFs in the three *B. forskalii* mitochondrial genomes. First, we verified that this test correctly predicted that low π*N*/π*S* values in invertebrate mitochondrial genomes were the result of negative selection (Table S6; Fig. S5), similar to previous results (Bazin, Glémin & Galtier, 2006). We found that the observed π*N*/π*S* values in URF1 and URF2 are below what is expected if the loci were evolving without selective pressure, with significant Monte Carlo *p*-values (Fig. S5). These results suggest that the higher π*N*/π*S* values for URF2 and URF1 relative to the canonical mitochondrial genes are due to less selective pressure, but not neutral evolution when measuring π*N*/π*S* over the complete loci. In biological terms, the putative protein products of URF1 and URF2 are evolutionarily constrained and may have biological function within the mitochondria.

### *Beroe forskalii* URFs have more nonsynonymous mutations in non-TM domains

The distributions of nonsynonymous and synonymous sites along the lengths of URF1 and URF2 suggest weaker selection in some regions of the ORFs, such as the portions of URF1 and URF2 predicted to be inside the mitochondrial matrix (Fig. S13; Tables S11–S14). However, the fact that all regions of URF1 and URF2 are under less selective pressure than canonical mitochondrial proteins, and the high accumulation of both synonymous and nonsynonymous mutations in the URFs, may suggests that URF1 and URF2 are less critical to *B. forskalii* mitochondrial function than the canonical genes.

### Ctenophore URFs appear to be mitochondrial TM transport proteins

The GO term and secondary structure predictions suggest that the *B. forskalii* URF1 and URF2 proteins, as well as all other known ctenophore mitochondrial URFs, are transmembrane transport proteins (See Supplemental GitHub/Zenodo Data and Table S3). Interestingly, the GO term predictions for all of the URFs from previously published ctenophores also hint at functions related to transmembrane transport. It is not possible to speculate on the transport substrate of URF1 and URF2 due to the high structural similarity of all transmembrane transport proteins and the lack of BLAST hits to public databases.

One hypothesis was that URF2 and URF1 are orthologs of the metazoan mitochondrially-encoded transmembrane protein ATP6, not present in other ctenophore mitogenomes. However, the presence of an ATP6 gene in the *B. forskalii* transcriptome indicates that it is nuclearly-encoded, as has been shown in *M. leidyi* and *P. bachei* (Pett et al., 2011; Kohn et al., 2012).

While the *B. forskalii* URFs and other ctenophore URFs appear to be exclusively transmembrane proteins with up to nine TM domains, one of the only known bilaterian mitochondrial URFs (from the brachiopod *Lingula*) appears to be a duplicated protein (Endo et al., 2005) with a maximum of two TM domains. Given this collection of evidence it is clear that URF1 and URF2, if truly protein coding, are genes that have not been characterized in other mitochondrial genomes. In the metazoa, the presence of additional mitochondrial protein-coding genes aside from the canonical thirteen is a trait only found in some Cnidarians and Poriferans (Gissi, Iannelli & Pesole, 2008). These findings mean that both the Ctenophora and the Porifera share the same set of structural genomic features as defined by Gissi, Iannelli & Pesole (2008): additional proteins, high gene rearrangement variability, all genes encoded in one direction, and a single coding strand. While mitochondrial phylogenetics have not been able to resolve early metazoan evolutionary relationships (Osigus et al., 2013; Bernt et al., 2013a), future studies of the additional proteins present in ctenophore and sponge mitogenomes may give clues to how their metabolic pathways evolved after diverging.

## Conclusion

There is a large body of work on bilaterian mitochondrial genomes, and among those mitogenomes there are few deviations from the canonical composition of thirteen protein coding genes, two ribosomal RNAs, and twenty two tRNAs. Non-bilaterian metazoan species, however, often have deviations from the canonical gene content, including poorly understood unidentified URFs. Here, we provided evidence that all sequenced ctenophore mitochondrial genomes contain URFs that appear to encode transmembrane transport proteins, and that the URFs in the mitochondrial genome of the ctenophore *B. forskalii* are under negative selection, and therefore are translated and functional within the mitochondria.

To confirm that these ctenophores contain the protein products of mitochondrial URFs, a future study may benefit from performing mass spectrometry experiments on purified fractions of ctenophore mitochondria to attempt to identify the protein products of the URFs. In addition, the sequencing of more ctenophore mitochondrial genomes at the population-level may reveal patterns that inform us of the “hidden biology” of ctenophores, their metabolism, and how they have adapted to diverse marine habitats.

## Supplemental Information

10.7717/peerj.8356/supp-1 Supplemental Information 1 Supplemental methods, figures, and tables.

## Additional Information and Declarations

### Field Study Permissions

The following information was supplied relating to field study approvals (i.e., approving body and any reference numbers): All ctenophore samples were collected under the State of California Department of Fish and Wildlife scientific collecting permit SC-4029.

### DNA Deposition

The following information was supplied regarding the deposition of DNA sequences: The mitochondrial genome sequences, paired-end reads, ONT reads, and ATP6 sequences supporting the conclusions of this article are available through the NCBI BioProject Portal at PRJNA421807.

### Data Availability

The following information was supplied regarding data availability: Additional copies of the mitochondrial and ATP6 sequences, I-TASSER results, BLAST results, and a snakemake pipeline to reproduce the figures and analyses in this manuscript can be found here: DOI 10.5281/zenodo.2678159. The pauvre software can be found at https://github.com/conchoecia/pauvre and the cuttlery software at https://github.com/conchoecia/cuttlery.
