# Supplementary material for: Conserved novel ORFs in the mitochondrial genome of the ctenophore Beroe forskalii
Source: PeerJ. 2020 Jan 27;8:e8356. doi: 10.7717/peerj.8356 (PMC6991124; doi:10.7717/peerj.8356)
Supplement: Supplemental Information 1 [file peerj-08-8356-s001.pdf]

# Supplemental Materials to

## Conserved novel ORFs in the mitochondrial genome of the ctenophore *Beroe forskalii*.

**Darrin T. Schultz [1,3,*], Jordan M. Eizenga [1], Russell B Corbett-Detig [1], Warren R Francis [2], Lynne M Christianson [3], Steven HD Haddock [3,4]**

[1]Department of Biomolecular Engineering and Bioinformatics, University of California Santa Cruz, 1156 High Street, Santa Cruz, California 95064, United States. [2]Department of Biology, University of Southern Denmark, Campusvej 55, Odense 5230, Denmark. [3]Monterey Bay Aquarium Research Institute, 7700 Sandholdt Road, Moss Landing, California 95039, United States. [4]Department of Ecology and Evolutionary Biology, University of California Santa Cruz, 1156 High Street, Santa Cruz, California 95064, United States.

## SUPPLEMENTAL MATERIALS

This section contains tables and figures not contained in the main body of the manuscript.


| | | | | |
|---|---|---|---|---|
| Page 2 | - | Methods | - | NDPS textual explanation. |
| Page 2 | - | Figure S-1 | - | NDPS explanation figure. |
| Page 3 | - | Methods | - | Bayesian hypothesis test calculations. |
| Page 4 | - | Text | - | Additional files descriptions. |
| Page 7 | - | Table S-1 | - | Sample Collection Information. |
| Page 7 | - | Figure S-2 | - | Sample Collection map. |
| Page 8 | - | Figure S-3 | - | Dotplot of the raw canu contig. |
| Page 9 | - | Table S-2 | - | Mitochondrial Insertions and deletions |
| Page 9 | - | Figure S-4 | - | Codon usage distributions |
| Page 10 | - | Table S-3 | - | PSIPRED results. |
| Page 11 | - | Table S-4 | - | Clustal Omega MSA results. |
| Page 12 | - | Table S-5 | - | Samples used in nucleotide diversity measures. |
| Page 13 | - | Figure S-5 | - | NDPS plots for non-*Beroe* species. |
| Page 14 | - | Table S-6 | - | Tabular results for non-*Beroe* NDPS. |
| Page 15 | - | Figure S-6 | - | Fourier Transform of *B. f.* mitogenomes. |
| Page 16 | - | Table S-7 | - | Table of samples used in the phylogenies. |
| Page 17 | - | Figure S-7 | - | Phylobayes phylogeny. All sites. |
| Page 18 | - | Figure S-8 | - | Phylobayes phylogeny, using Guidance2. |
| Page 19 | - | Figure S-9 | - | RAxML phylogeny. All sites. |
| Page 20 | - | Figure S-10 | - | RAxML phylogeny, using Guidance2. |
| Page 21 | - | Figure S-11 | - | All trees, side-by-side. |
| Page 22 | - | Table S-8 | - | Phylobayes summary statistics, pt. 1. |
| Page 22 | - | Table S-9 | - | Phylobayes summary statistics, pt. 2. |
| Page 22 | - | Table S-10 | - | Phylobayes summary statistics, pt. 3. |
| Page 23 | - | Table S-11 | - | Counts of synonymous and nonsynonymous sites. |
| Page 23 | - | Table S-12 | - | Total number of sites of type TM, IM, or MM. |
| Page 24 | - | Table S-13 | - | Percent of each domain that has N or S sites. |
| Page 24 | - | Table S-14 | - | Percent of all sites of type N or S. |
| Page 25 | - | Figure S-12 | - | The FACIL codon logo. |
| Page 26 | - | Table S-15 | - | The table of FACIL results. |
| Page 27 | - | Figure S-13 | - | Plot of nucleotide diversity along genes. |


[*] To whom correspondence should be addressed. Email: dts@ucsc.edu

# SUPPLEMENTAL METHODS

## Supplemental Methods - Nucleotide Diversity Permutation Simulation

In simple terms, the nucleotide diversity permutation simulation (NDPS) randomly chooses one of the input sequences, then randomly mutates it to simulate neutral evolution. The $\pi N/\pi S$ value for these sequences is measured, then the process is repeated.

The NDPS algorithm can be broken down into four steps: In step one, the ORFs are aligned, and the between-individual mutation profile is recorded (Fig. S-1).

In step two, one of the input sequences is randomly chosen, and the remaining sequences are mutated at randomly selected alignment columns using mutation profile recorded in the first step (See Fig. S-1 for a visual explanation). The mutation profile ensures that the simulations are also constrained such that the simulated sequences share the same genealogy of the original sequences. This genealogical constraint corrects for variations in estimated $\pi N/\pi S$ caused by using the NG method with three or more sequences of different levels of divergence, as described in Yang and Nielsen (2000). We used a uniform distribution for possible mutations, so on average the mutated sequences would share the same transition and transversion mutation rates - another source of error in the NG method (Yang and Nielsen, 2000). Simulations that contained nonsense mutations were thrown out.

In step three, the new $\pi N/\pi S$ value of the sequence alignment is calculated. Here also we used the NG nonsynonymous-conservative method implemented in biopython's cal_dn_ds function (Nei and Gojobori, 1986; Cock et al., 2009). For each locus we generated 1000 sets of genealogically-identical mutated sequence sets per locus to build a distribution of $\pi N/\pi S$ values.

In step four we computed the Monte-Carlo $p$-value for the probability that each ORF/URF is the result of an evolutionary process with no selection. The Monte Carlo p-value is the quantity of simulations falling below the observed $\pi N/\pi S$ value divided by the total number of simulations for which $\pi S > 0$ (North et al., 2002). This constraint makes the test more conservative when looking for evidence of negative selection. Note that the Nei-Gojobori (1986) method of estimating $\pi N/\pi S$ conservatively estimates the number of nonsynonymous mutations, indicating that the means of the NDP simulation may actually be preferentially shifted toward zero. This makes the upper limit of our Monte-Carlo p-value more stringent.

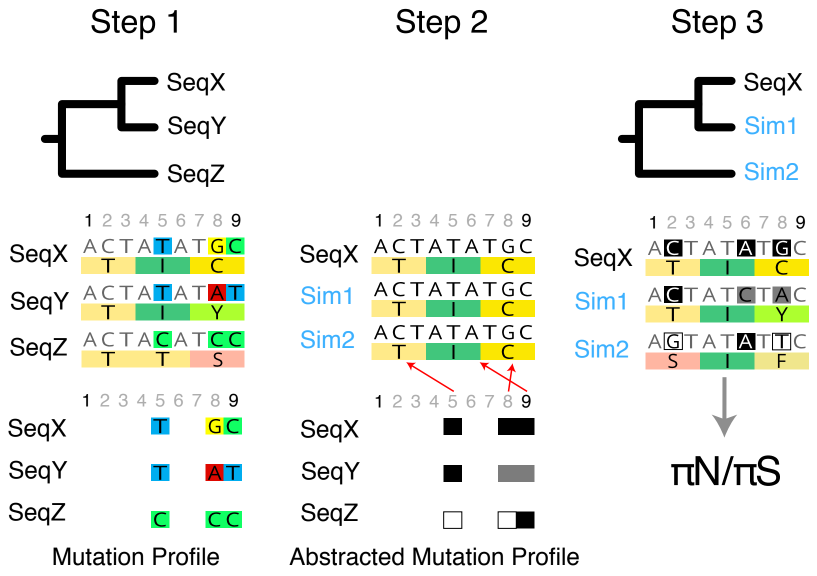


**Figure S-1.** ***Nucleotide Diversity Permutation Simulation*** In step 1 a mutation profile is built from an ORF alignment. In step two one of the sequences is chosen, then mutated at random positions (red arrows) according to the mutation profile. In step three $\pi N/\pi S$ is calculated from the new sequence set. The use of the mutation profile ensures that the phylogenetic relationship of the mutated sequences is the same as the phylogenetic relationship of the input sequences.

**Supplemental Methods - Bayesian hypothesis test calculations**

We assume that sequences are generated from one of two distributions, one corresponding to ORFs, the other to non-coding sequence. For this analysis, sequences are assumed to be vectors of length 64 that contain the count of codons.

$$x_{ORF}|\theta_{ORF}, N \sim F_{ORF} = \text{Multi}(\theta_{ORF}, N) \tag{1}$$
$$x_{NC}|\theta_{NC}, N \sim F_{NC} = \text{Multi}(\theta_{NC}, N) \tag{2}$$

We also assume that the length of a sequence $N$ is uninformative. Accordingly, it is ignored in subsequent analysis. The parameters for the ORF and non-coding distributions are assumed to share a equivalent but unshared Dirichlet prior. Note that here we use the Dirichlet prior of 0.5. We have also tried 1 as the prior with no noticeable outcome on the results of the test.

$$\theta_{ORF}, \theta_{NC} \sim \text{Dirichlet}(1/2, \dots, 1/2) \tag{3}$$

Our data consist of a set of sequences $X_{ORF}$ known to be ORFs, a set of sequences $X_{NC}$ known to be non-coding, and a single sequence $x$ which may be in either group. This yields the following hypotheses:

$$H_1 : x \sim F_{ORF}|X_{ORF} \tag{4}$$
$$H_0 : x \sim F_{NC}|X_{NC} \tag{5}$$

Let $n_{i,ORF}$ be the count of codon $i$ in $X_{ORF}$. The posterior predictive likelihood of $x$ under $H_1$ is then

$$P(x|H_1, X_{ORF}) = \int_{\theta_{ORF}} P(x|\theta_{ORF}, X_{ORF}) P(\theta_{ORF}|X_{ORF}) d\theta_{ORF} \tag{6}$$
$$= \binom{\sum_i x_i}{x_1 \dots x_{64}} \frac{\Gamma\left(\sum_i (1/2 + n_{i,ORF})\right)}{\prod_i \Gamma(1/2 + n_{i,ORF})} \frac{\prod_i \Gamma(1/2 + n_{i,ORF} + x_i)}{\Gamma\left(\sum_i (1/2 + n_{i,ORF} + x_i)\right)} \tag{7}$$

Similarly,

$$P(x|H_0, X_{NC}) = \binom{\sum_i x_i}{x_1 \dots x_{64}} \frac{\Gamma\left(\sum_i (1/2 + n_{i,NC})\right)}{\prod_i \Gamma(1/2 + n_{i,NC})} \frac{\prod_i \Gamma(1/2 + n_{i,NC} + x_i)}{\Gamma\left(\sum_i (1/2 + n_{i,NC} + x_i)\right)} \tag{8}$$

Taking $X$ to indicate the entire data set, the final posterior odds is given by

$$\frac{P(H_1|X)}{P(H_0|X)} = \frac{P(x|H_1, X_{ORF})}{P(x|H_0, X_{NC})} \frac{P(H_1)}{P(H_0)} \tag{9}$$

The second term is the prior odds in favor of the ORF hypothesis. It can be taken to be 1 for an unbiased test.

**Additional Files in the Zenodo Data.**

- This repository contains additional files for the manuscript, ”Novel ORFs in the mitochondrial genome of the ctenophore, Beroe forskalii”
- To recreate most of the figures for the manuscript, please install `snakemake`, `cuttlery`, and `pauvre`, then naviagate to this directory and run the snakemake pipeline by executing the command `snakemake` in your terminal.

# 1 ABBREVIATIONS

- *Pb* - *Pleurobrachia bachei*
- *Ml* - *Mnemiopsis leidyi*
- *Bf* - *Beroe forskalii*

# 2 ADDITIONAL FILES

## 2.1 Directory – `16S_structure`

Files in this directory are related to determining the 16S structure of the *B. forskalii* mitochondrial genome. The files in this directory are:

- `mnemiopsis_rrnl_final.sto` is a structural Stockholm file. This encodes the *M. leidyi* 16S rRNA structure from Pett et al 2011.
- `mnemi16S.cm` is the infernal covariance model built using `mnemiopsis_rrnl_final.sto`.
- `Bf1311_against_mnemi16S.txt` is the infernal results file when the Bf1311 mitochondrial genome was searched against using the `mnemi16S.cm` covariance model.

## 2.2 Directory – `ARWEN`

This directory contains the fasta files of each *B. forskalii* mitochondrial genome and the ARWEN results. The files in this directory are:

- `MG655622.fasta`- The Bf201706 mitochondrial genome.
- `MG655622_results.txt` - The Bf201706 ARWEN results.
- `MG655623.fasta` - The Bf201606 mitochondrial genome.
- `MG655623_results.txt` - The Bf201606 ARWEN results.
- `MG655624.fasta`- The Bf201311 mitochondrial genome.
- `MG655624_results.txt` - The Bf201311 ARWEN results.

## 2.3 Directory – `ATP6`

Files in this directory pertain to ATP6 of all ctenophores. This directory contains:

- `README.md` contains notes about where to locate the *P. bachei* and *M. leidyi* ATP6 sequences.
- `PB_ML_ATP6_nucl.fasta` contains the *Pb* and *Ml* ATP6 transcript DNA sequences.
- `PB_ML_ATP6_prot.fasta` contains the *Pb* and *Ml* ATP6 protein sequences.
- `ATP6_to_BF.txt` contains the tblastn results using the *Pb* and *Ml* ATP6 sequences to query the *Bf* transcriptome
- `BF_ATP6_hits.fasta` contains the transcript sequences of the *Bf* ATP6 blast hits.
- `BF_ATP6.fasta` contains the most likely *Bf* ATP6 transcript based on protein sequence similarity to other ctenophore ATP6 sequences.
- `DS12*/DS12*_mapdepthavg.txt` contains the average map depth average when the DS121 and DS122 libraries were mapped against the *B. forskalii* ATP6 transcript using `bwa mem`.

## 2.4 Directory – `Biosample_accessions`

Text files in this directory contain the NCBI BioSample Accession numbers for all four *B. forskalii* ctenophore individuals.

## 2.5 Directory – `CREx`

This directory contains the file, `crex_results_summary.pdf`, which are the CREx mitochondrial rearrangement analysis results for the *M. leidyi*, *B. forskalii*, and *P. bachei* mitochondrial genomes.

## 2.6 Directory – FTGwindow

This directory contains files used in the Fourier Transform analysis to predict which regions of the mitochondrial genome contain protein-coding DNA.

## 2.7 Directory – assembly

This directory contains a single file, `bf_raw_mito.fa`, which is the raw mitochondrial genome assembly produced by canu.

## 2.8 Directory – fasta_sequences

This directory contains fasta files used in various analyses, including nucleotide and amino acid sequences, as well as various alignments. The files in this directory are

- Directory `BF201706_prot`
- Directory `TM_results`
  - contains html file results from TMHMM for COX1, COX2, COX3, CYTB, ND1-6, URF1, and URF2.
- Directory `TM_txtfiles`
  - Contains text files with transmembrane domain predictions by TMHMM. There are files for COX1, COX2, COX3, CYTB, ND1-6, URF1, and URF2.
- file `Bf201706_prot.fasta` - the protein sequences from MG655622/Bf201706. These were used in generating the transmembrane domain prediction with TMHMM.
- Directory `alignments`
- Directory `concatenated_after_guidance`
  - `concatenated_prot.phy` is the COX1, COX2, COX3, CYTB, ND1, ND3, and ND5 alignments concatenated together. These are the protein alignments that have had sites removed using Guidance2.
- Directory `concatenated_noguidance`
  - `concatenated_noguidance.phy` is the COX1, COX2, COX3, CYTB, ND1, ND3, and ND5 alignments concatenated together. No columns were removed using Guidance2.
- Directory `ctenos_all_proteins_noguidance`
  - `all_proteins_ctenos_monoallo_noguidance.phy` is the concatenated alignment for COX1, COX2, COX3, CYTB, and ND1-6 for all ctenophores and two outgroups.
- Directory `guidance_alignments` - This directory contains a files and script, `run_guidance.sh`, that produces alignments with columns removed using Guidance2.
- Directory `prot_cteno_aln` contains nucleotide alignments for 12S and 16S for all ctenophore mitochondrial genomes, as well as protein alignments for all ctenophores for genes COX1, COX2, COX3, CYTB, and ND1-ND6.
- file `12S.fasta` - 12S alignment from *Pb* and other ctenophores.
- file `16S.fasta` - 16S alignment from *Bf* and other ctenophores.
- Directory `coding_seqs` contains all of the nucleotide sequences for *Bf* for COX1, COX2, COX3, CYTB, and ND1-6.
- Directory `non-beroe` contains directories of nucleotide sequences for coding and noncoding regions of the following organisms: *Chlamydomonas*, *Daphnia*, *Drosophila*, Human, and *Strongylocentrotus*.
- Directory `noncoding_seqs` contains all of the *Bf* nucleotide sequences for the noncoding regions `COX1 to ND6`, `COX3 to ND3`, `ND2 to CYTB`, `ND5 to URF1`, `URF1 to URF2`, and `URF2 to ND2`.
- Directory `test_seqs` contains all of the *Bf* nucleotide sequences for URF1 and URF2.
- file `bf_mitogenomes_alignment.fasta` - the whole-mitogenome *Bf* alignment used to generate the table listing indels.

## 2.9 Directory – figures

When the `snakemake` pipeline is run, the figures and associated text files are output to this directory.

### 2.10 Directory – `final_annotations`

Text files in this directory include the final DNA sequences of the mitochondrial genomes of individuals Bf1311, Bf1706, and Bf1606. In addition, we include the scripts `map_depth_extract.sh` and `FastqPairedEndValidator.pl` used to isolate genomic reads that map to the mitochondrial sequences.

### 2.11 Directory – `gff_files`

This directory contains GFF files used in plotting mitochondrial genomes for synteny.

### 2.12 Directory – `indels`

Contains scripts and files to analyze the number and distribution of indels between individuals.

- file `Bf_alignment.fasta` is a whole-mitogenome alignment for all three individuals of *B. forskalii*
- file `Bf_alignment.geneious` - the same alignment, in geneious format.
- file `Bforsk_indels.txt` - a table of indels, the sample in which they occur, the position, and the size.
- file `print_gaps.py` - a python script that produces `Bforsk_indels.txt` from `Bf_alignment.fasta`

### 2.13 Directory – `itasser_results`

Text and HTML files in this directory are from the ITASSER protein structure prediction. Additionally there are structure files that can be opened with protein viewing software.

### 2.14 Directory - `phylogeny`

Files in this directory contain phylogenetic analyses. All subdirectories listed below are in the directory `phylogeny/201904_rooted_tree`.

- Directory `RAxML_ctenos_allgenes_noguidance` - RAxML analysis conducted on COX1, COX2, COX3, CYTB, and ND1-6 using only ctenophores with two outgroups. Guidance2 was not used to remove columns from the amino acid matrix.
- Directory `RAxML_protcatwag_guidance` - RAxML analysis conducted on COX1, COX2, COX3, CYTB, ND1, ND3, and ND5 using ctenophores and many outgroups. Guidance2 **was** used to remove columns from the amino acid matrix.
- Directory `RAxML_protcatwag_noguidance` - RAxML analysis conducted on COX1, COX2, COX3, CYTB, ND1, ND3, and ND5 using ctenophores and many outgroups. Guidance2 **was not** used to remove columns from the amino acid matrix.
- Directory `phylobayes_ctenos_allgenes_noguidance` - Phylobayes analysis conducted on COX1, COX2, COX3, CYTB, and ND1-6 using only ctenophores with two outgroups. Guidance2 was not used to remove columns from the amino acid matrix.
- Directory `phylobayes_guidance` - Phylobayes analysis conducted on COX1, COX2, COX3, CYTB, ND1, ND3, and ND5 using ctenophores and many outgroups. Guidance2 **was** used to remove columns from the amino acid matrix.
- Directory `phylobayes_noguidance` - Phylobayes analysis conducted on COX1, COX2, COX3, CYTB, ND1, ND3, and ND5 using ctenophores and many outgroups. Guidance2 **was not** used to remove columns from the amino acid matrix.

### 2.15 Directory - `tRNAscanSE`

Contains HTML files of results from running tRNAscanSE on the whole mitochondrial genomes for Bf201706, Bf201606, and Bf201311.

| Sample Name | type | Lat | Lon | Depth (m) | Date Collected | Library names |
|---|---|---|---|---|---|---|
| Bf201311 | DNA | 36°23’5”N | 122°40’3”W | BWD | 2013-11-23 | 10673X1 |
| Bf201606 | DNA | 35°55’56”N | 122°55’58”W | BWD | 2016-06-12 | DS117, DS118 |
| Bf201706 | DNA | 35°56’4”N | 122°55’37”W | BWD | 2017-06-08 | DS136 |
| Bf201507 | RNA | 36°35’56”N | 122°9’8”W | 400 | 2015-07-13 | DS121, DS122 |

**Table S-1.** Collection dates, coordinates, collection methodology, and resulting library names for each sample used in this study. BWD indicates a sample caught during a blue water dive, or a depth between 0-30 meters. Mitochondrial reads are available on SRA at https://www.ncbi.nlm.nih.gov/bioproject/PRJNA421807

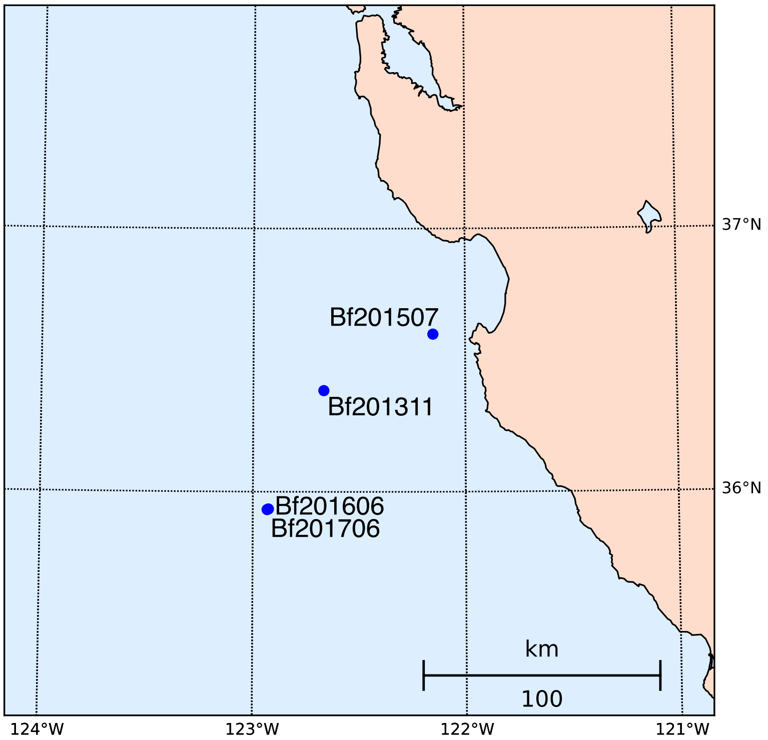


**Figure S-2.** **Map of sample collection locations.**

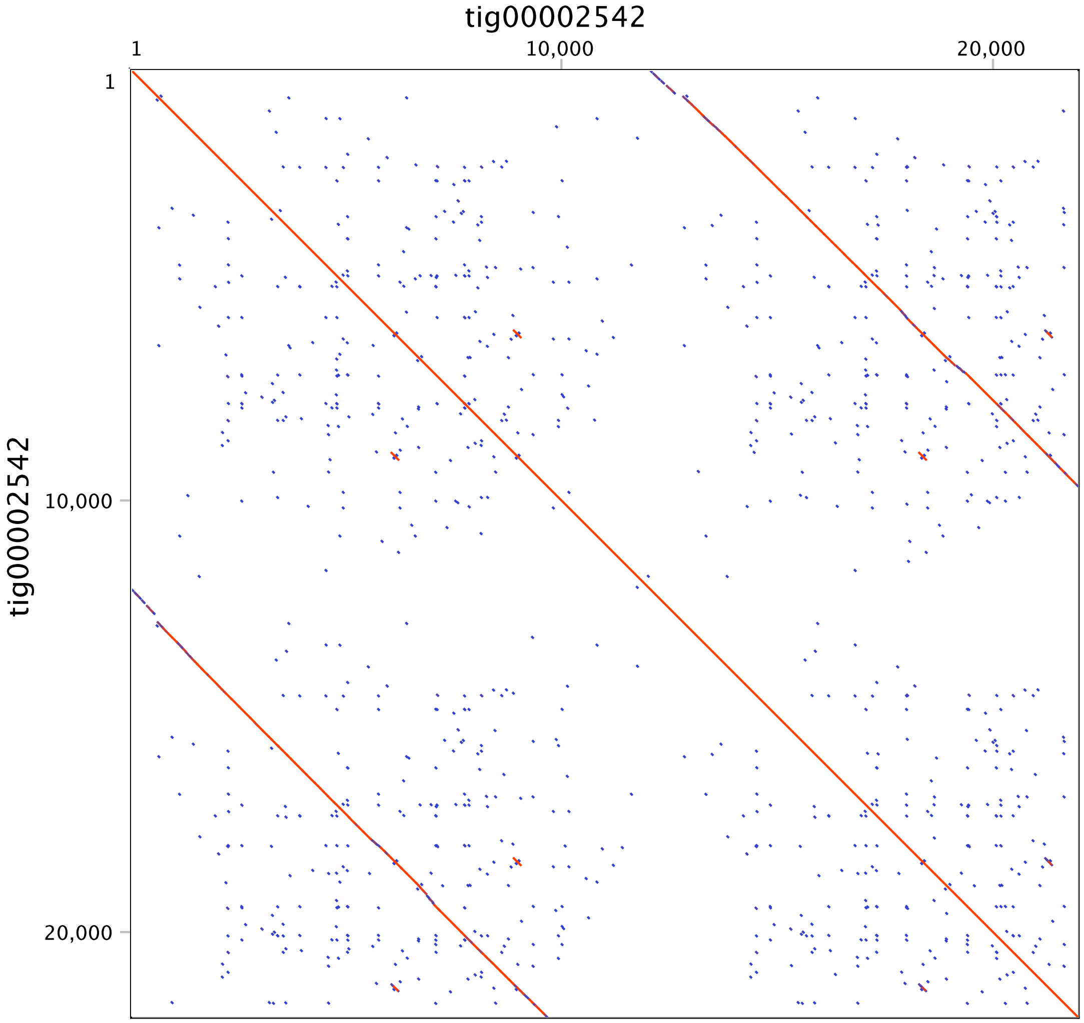


**Figure S-3. Dotplot of raw canu contig.** This figure was created using a word size of 15. This dotplot depicts two concatenated copies of the mitochondrial genome as assembled by canu.

| Record | Gap Start | Gap Length |
|---|---|---|
| MG655624_Bf201311 | 4106 | 2 |
| MG655624_Bf201311 | 4114 | 2 |
| MG655624_Bf201311 | 4259 | 2 |
| MG655624_Bf201311 | 8843 | 1 |
| MG655624_Bf201311 | 10042 | 2 |
| MG655623_Bf201606 | 4106 | 2 |
| MG655623_Bf201606 | 4231 | 1 |
| MG655623_Bf201606 | 4319 | 4 |
| MG655623_Bf201606 | 7595 | 16 |
| MG655623_Bf201606 | 8876 | 1 |
| MG655623_Bf201606 | 8948 | 1 |
| MG655623_Bf201606 | 9227 | 1 |
| MG655623_Bf201606 | 10043 | 1 |
| MG655622_Bf201706 | 4203 | 1 |
| MG655622_Bf201706 | 4259 | 2 |
| MG655622_Bf201706 | 8704 | 12 |
| MG655622_Bf201706 | 8720 | 9 |
| MG655622_Bf201706 | 8842 | 2 |
| MG655622_Bf201706 | 8876 | 1 |
| MG655622_Bf201706 | 9227 | 1 |

**Table S-2.** Insertions and deletions in the alignment of the three *B. forskalii* mitochondrial genomes.

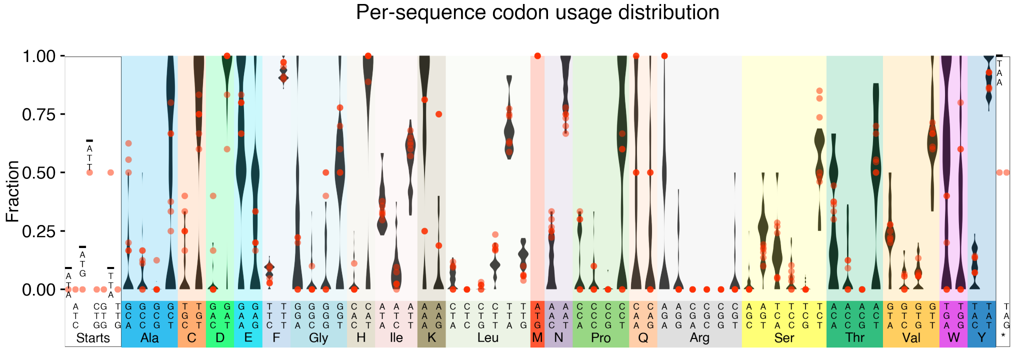


**Figure S-4. Mitochondrial Codon Usage Distribution Table.** The codon usage frequency of the canonical genes are plotted as violin plots. The data comprising the violin plots are the codon usage of each gene from each *B. forskalii* individual. The red dots overlaid on the codon usage plot are the codon usage frequencies of URF2 and URF1 from different *B. forskalii* individuals.

| Species | URF | AA count | TM domain count | Top three Biological Process GO terms (Prob) | Top three Molecular Function GO terms (Prob) |
|---|---|---|---|---|---|
| *B. forskalii* | 1 | 354 | 9 | GO:0098655 cation transmembrane transport (0.879)<br>GO:0006810 transport (0.859)<br>GO:0007166 cell surface receptor signaling pathway (0.840) | GO:0003824 catalytic activity (0.959)<br>GO:0022857 transmembrane transporter activity (0.951)<br>GO:0005215 transporter activity (0.932) |
| *B. forskalii* | 2 | 222 | 7 | GO:0006810 transport (0.864)<br>GO:0055085 transmembrane transport (0.774)<br>GO:0050877 neurological system process (0.772) | GO:0003824 catalytic activity (0.890)<br>GO:0022857 transmembrane transporter activity (0.875)<br>GO:0005215 transporter activity (0.860) |
| *P. bachei* | 1 | 37 | 1 | GO:0019222 regulation of metabolic process (0.958)<br>GO:0006810 transport (0.889)<br>GO:0006811 ion transport (0.888) | GO:0008270 zinc ion binding (0.986)<br>GO:0016301 kinase activity (0.972)<br>GO:0005125 cytokine activity (0.961) |
| *P. bachei* | 2 | 42 | 1 | GO:0019222 regulation of metabolic process (0.980)<br>GO:0034220 ion transmembrane transport (0.889)<br>GO:0006810 transport (0.854) | GO:0005125 cytokine activity (0.954)<br>GO:0016817 hydrolase activity, acting on acid anhydrides (0.900)<br>GO:0003824 catalytic activity (0.862) |
| *P. bachei* | 3 | 45 | 1 | GO:0019222 regulation of metabolic process (0.967)<br>GO:0034645 cellular macromolecule biosynthetic process (0.873)<br>GO:0006810 transport (0.862) | GO:0005125 cytokine activity (0.927)<br>GO:0022857 transmembrane transporter activity (0.848)<br>GO:0005215 transporter activity (0.840) |
| *M. leidyi* | 1 | 46 | 2 | GO:0019222 regulation of metabolic process (0.976)<br>GO:0006810 transport (0.826)<br>GO:0007166 cell surface receptor signaling pathway (0.718) | GO:0008270 zinc ion binding (0.921)<br>GO:0032549 ribonucleoside binding (0.885)<br>GO:0003676 nucleic acid binding (0.751) |
| *V. multiformis* | 1 | 27 | | too short for PSIPRED | too short for PSIPRED |
| *C. loyai* | 1 | 228 | 7 | GO:0006810 transport (0.875)<br>GO:0098655 cation transmembrane transport (0.848)<br>GO:0055085 transmembrane transport (0.800) | GO:0022857 transmembrane transporter activity (0.941)<br>GO:0005215 transporter activity (0.929)<br>GO:0022891 substrate-specific transmembrane transporter activity (0.903) |
| *C. loyai* | 2 | 36 | 1 | GO:0019222 regulation of metabolic process (0.938)<br>GO:0006810 transport (0.870)<br>GO:0006812 cation transport (0.833) | GO:0001882 nucleoside binding (0.977)<br>GO:0016301 kinase activity (0.959)<br>GO:0032549 ribonucleoside binding (0.956) |
| *C. yulianicorum* | 1 | 93 | 3 | GO:0006810 transport (0.878)<br>GO:0015672 monovalent inorganic cation transport (0.864)<br>GO:0034645 cellular macromolecule biosynthetic process (0.847) | GO:0022890 inorganic cation transmembrane transporter activity (0.886)<br>GO:0005215 transporter activity (0.837)<br>GO:0022857 transmembrane transporter activity (0.823) |
| *C. yulianicorum* | 2 | 106 | 2 | GO:0050911 detection of chemical stimulus involved in sensory perception of smell (0.971)<br>GO:0034645 cellular macromolecule biosynthetic process (0.948)<br>GO:0034220 ion transmembrane transport (0.946) | GO:0022857 transmembrane transporter activity (0.919)<br>GO:0003824 catalytic activity (0.913)<br>GO:0008324 cation transmembrane transporter activity (0.910) |
| *C. yulianicorum* | 3 | 65 | 2 | GO:0019222 regulation of metabolic process (0.933)<br>GO:0055085 transmembrane transport (0.884)<br>GO:0007166 cell surface receptor signaling pathway (0.880) | GO:0005125 cytokine activity (0.895)<br>GO:0022890 inorganic cation transmembrane transporter activity (0.882)<br>GO:0015077 monovalent inorganic cation transmembrane transporter activity (0.869) |
| *C. yulianicorum* | 4 | 46 | 1 | GO:0019222 regulation of metabolic process (0.881)<br>GO:0006810 transport (0.848)<br>GO:0007166 cell surface receptor signaling pathway (0.797) | GO:0016817 hydrolase activity, acting on acid anhydrides (0.943)<br>GO:0001882 nucleoside binding (0.882)<br>GO:0032549 ribonucleoside binding (0.873) |

**Table S-3. PSIPRED results** This table contains the transmembrane domain count as predicted by MEMSAT-SVM, and the top Biological Process and Molecular Function GO terms (McGuffin et al., 2000). Many of the URFs have biological and molecular functions related to transmembrane transport.

| Species | URF | AA count | | | | | | | | | | | | | |
|---|---|---|---|---|---|---|---|---|---|---|---|---|---|---|---|
| *Bf* | 1 | 354 | 100 | | | | | | | | | | | | |
| *Bf* | 2 | 222 | 27.8 | 100 | | | | | | | | | | | |
| *Pb* | 1 | 37 | 21.6 | 16.1 | 100 | | | | | | | | | | |
| *Pb* | 2 | 42 | 20.6 | 14.3 | | 100 | | | | | | | | | |
| *Pb* | 3 | 45 | 24.1 | | | 8.3 | 100 | | | | | | | | |
| *Ml* | 1 | 46 | 30.4 | 22.0 | 31.4 | | | 100 | | | | | | | |
| *Vm* | 1 | 27 | 29.6 | 23.1 | 28.6 | | | 33.3 | 100 | | | | | | |
| *Cl* | 1 | 228 | 24.2 | 30.6 | | 22.9 | 27.5 | 38.1 | 37.5 | 100 | | | | | |
| *Cl* | 2 | 36 | 20.0 | 44.4 | 50.0 | 25.0 | | 28.6 | 23.1 | 40.0 | 100 | | | | |
| *Cy* | 1 | 93 | 30.0 | | | 22.2 | 18.8 | | | 53.9 | | 100 | | | |
| *Cy* | 2 | 106 | 25.3 | 29.4 | | | 25.0 | | | 42.9 | | | 100 | | |
| *Cy* | 3 | 65 | 29.7 | 28.1 | 16.0 | | | 30.6 | 33.3 | 53.1 | 33.3 | | 23.5 | 100 | |
| *Cy* | 4 | 46 | 18.2 | 40.0 | 30.0 | 25.0 | | 26.7 | 14.3 | 41.2 | 83.3 | | | 41.2 | 100 |
| | | | 354 | 222 | 37 | 42 | 45 | 46 | 27 | 228 | 36 | 93 | 106 | 65 | 46 |
| | | | *Bf* | *Bf* | *Pb* | *Pb* | *Pb* | *Ml* | *Vm* | *Cl* | *Cl* | *Cy* | *Cy* | *Cy* | *Cy* |
| | | | 1 | 2 | 1 | 2 | 3 | 1 | 1 | 1 | 2 | 1 | 2 | 3 | 4 |

**Table S-4. Clustal Omega MSA results** This is a protein percent identity matrix. For example, *P. bachei* URF1 is 16.1% similar to *B. forskalii* URF2. Cells with no value had no alignments passing quality filter in clustal. The longest URF's AA sequences typicall

| NCBI | Genus | Species | citation |
|---|---|---|---|
| EU306621 | Chlamydomonas | reinhardtii | (Smith and Lee, 2008) |
| EU306619 | Chlamydomonas | reinhardtii | (Smith and Lee, 2008) |
| EU306617 | Chlamydomonas | reinhardtii | (Smith and Lee, 2008) |
| EU306622 | Chlamydomonas | reinhardtii | (Smith and Lee, 2008) |
| EU306623 | Chlamydomonas | reinhardtii | (Smith and Lee, 2008) |
| MH683671 | Daphnia | magna | |
| MH683670 | Daphnia | magna | |
| MH683669 | Daphnia | magna | |
| MH683668 | Daphnia | magna | |
| MH683664 | Daphnia | magna | |
| MH683665 | Daphnia | magna | |
| MH683663 | Daphnia | magna | |
| MH683667 | Daphnia | magna | |
| MH683666 | Daphnia | magna | |
| KP843854 | Drosophila | melanogaster | (Wolff et al., 2016) |
| KP843853 | Drosophila | melanogaster | (Wolff et al., 2016) |
| KP843852 | Drosophila | melanogaster | (Wolff et al., 2016) |
| KP843851 | Drosophila | melanogaster | (Wolff et al., 2016) |
| KP843850 | Drosophila | melanogaster | (Wolff et al., 2016) |
| KP843849 | Drosophila | melanogaster | (Wolff et al., 2016) |
| KP843848 | Drosophila | melanogaster | (Wolff et al., 2016) |
| KP843847 | Drosophila | melanogaster | (Wolff et al., 2016) |
| KP843846 | Drosophila | melanogaster | (Wolff et al., 2016) |
| KP843845 | Drosophila | melanogaster | (Wolff et al., 2016) |
| KP843844 | Drosophila | melanogaster | (Wolff et al., 2016) |
| KP843843 | Drosophila | melanogaster | (Wolff et al., 2016) |
| KP843842 | Drosophila | melanogaster | (Wolff et al., 2016) |
| FJ986465 | Homo | sapiens | (Yang et al., 2009) |
| GU170820 | Homo | sapiens | (Rani et al., 2010) |
| GQ859272 | Homo | sapiens | (Guillet et al., 2010) |
| MG936625 | Homo | sapiens | (van de Loosdrecht et al., 2018) |
| DQ826448 | Homo | sapiens | |
| KY964300 | Strongylocentrotus | intermedius | |
| KC898198 | Strongylocentrotus | intermedius | (Kober and Bernardi, 2013) |
| NC023772 | Strongylocentrotus | intermedius | |
| KY964299 | Strongylocentrotus | intermedius | |

**Table S-5. Nucleotide diversity permutation test non-*Beroe* samples** These were the samples used for the nucleotide diversity mutation permutation test. The NCBI nucleotide accession number, species, and citation are provided.

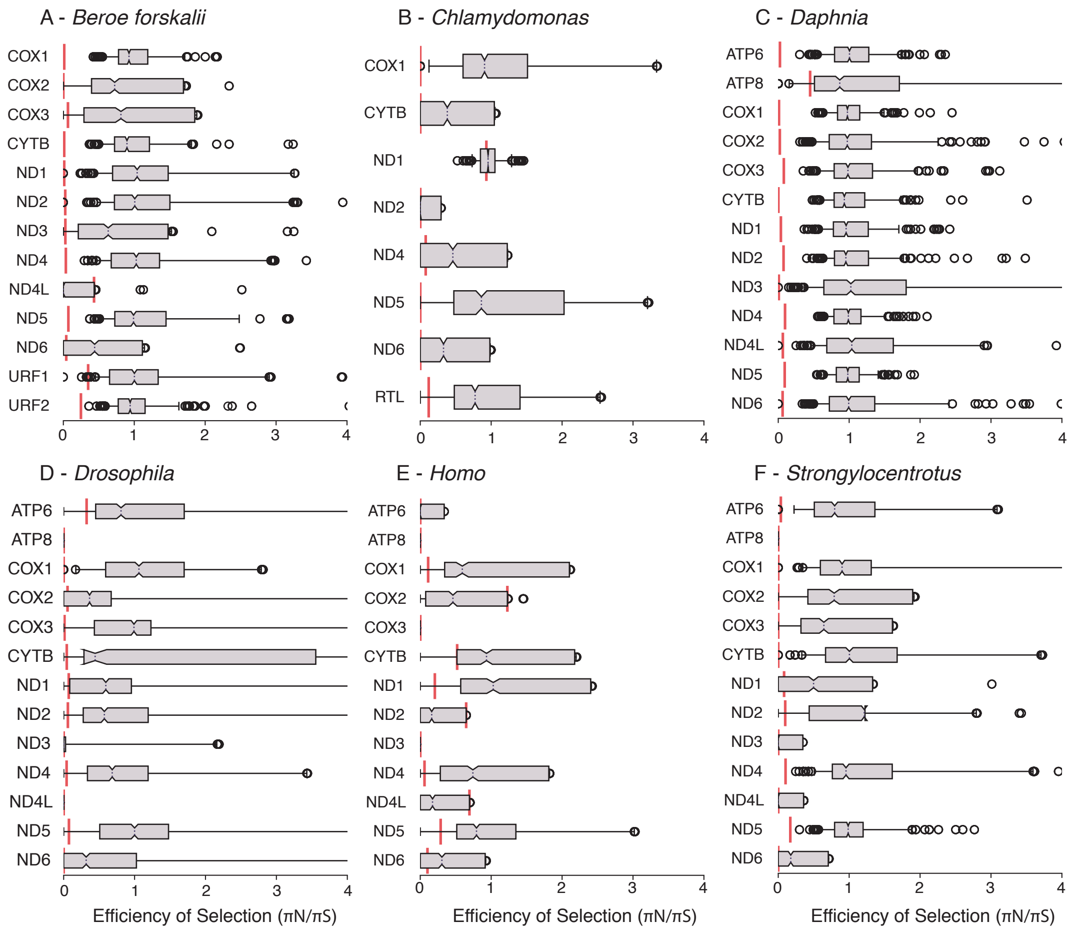


**Figure S-5. Nucleotide diversity permutation results** The results of performing the nucleotide diversity permutation test on and (A) three individuals of *B. forskalii*, (B) five individuals of *Chlamydomonas* (Smith and Lee, 2008), (C) nine individuals of *Daphnia*, (D) thirteen individuals of *Drosophila* (Wolff et al., 2016), (E) five individuals of human (Yang et al., 2009; Rani et al., 2010; Guillet et al., 2010; van de Loosdrecht et al., 2018), and (F) four individuals of *Strongylocentrotus* (Kober and Bernardi, 2013). Red vertical bars are the observed $\pi N/\pi S$ value for that locus, and the boxplots are of the $\pi N/\pi S$ distribution from the nucleotide diversity permutation simulation. The boxplot boxes extend from the lower to upper quartile values of the data. The median of the data is located at the notch. The notch width indicates the confidence interval around the median. The whiskers extend from the 5th to 95th percentile. Fliers are points falling outside of the 5th to 95th percentile. All ORFs and URFs, with the exceptions of ND4L and ND6, fall below or near the 5th percentile of mutation simulation $\pi N/\pi S$ values. In most loci in any given species, the observed $\pi N/\pi S$ values fall outside of or very low in the distribution of $\pi N/\pi S$ from simulated sequences. These results and figures were generated with cuttlery piNpiSsim.

| p_val | pi | piNpiS | seqname | species | genus |
|---|---|---|---|---|---|
| 0.013 | 0.001 | 0.077 | ND4 | Chlamydomonas | reinhardtii |
| 0.011 | 0.002 | 0.000 | CYTB | Chlamydomonas | reinhardtii |
| 0.001 | 0.002 | 0.119 | RTL | Chlamydomonas | reinhardtii |
| 0.000 | 0.001 | 0.000 | COX1 | Chlamydomonas | reinhardtii |
| 0.452 | 0.150 | 0.932 | ND1 | Chlamydomonas | reinhardtii |
| 0.000 | 0.001 | 0.000 | ND5 | Chlamydomonas | reinhardtii |
| 0.006 | 0.003 | 0.000 | ND6 | Chlamydomonas | reinhardtii |
| 0.171 | 0.001 | 0.000 | ND2 | Chlamydomonas | reinhardtii |
| 0.000 | 0.038 | 0.024 | ATP6 | Daphnia | magna |
| 0.000 | 0.014 | 0.096 | ND4 | Daphnia | magna |
| 0.000 | 0.014 | 0.091 | ND5 | Daphnia | magna |
| 0.000 | 0.025 | 0.022 | COX2 | Daphnia | magna |
| 0.000 | 0.034 | 0.061 | ND6 | Daphnia | magna |
| 0.000 | 0.021 | 0.011 | COX1 | Daphnia | magna |
| 0.142 | 0.087 | 0.449 | ATP8 | Daphnia | magna |
| 0.000 | 0.023 | 0.037 | ND1 | Daphnia | magna |
| 0.000 | 0.020 | 0.008 | ND3 | Daphnia | magna |
| 0.000 | 0.013 | 0.079 | COX3 | Daphnia | magna |
| 0.000 | 0.073 | 0.065 | ND4L | Daphnia | magna |
| 0.000 | 0.023 | 0.075 | ND2 | Daphnia | magna |
| 0.000 | 0.020 | 0.002 | CYTB | Daphnia | magna |
| 0.000 | 0.015 | 0.059 | ND2 | Drosophila | melanogaster |
| 0.012 | 0.009 | 0.055 | COX2 | Drosophila | melanogaster |
| 0.020 | 0.015 | 0.000 | ND3 | Drosophila | melanogaster |
| 0.000 | 0.006 | 0.044 | CYTB | Drosophila | melanogaster |
| 0.000 | 0.008 | 0.041 | ND4 | Drosophila | melanogaster |
| 0.000 | 0.012 | 0.012 | COX3 | Drosophila | melanogaster |
| 0.114 | 0.008 | 0.324 | ATP6 | Drosophila | melanogaster |
| 0.000 | 0.018 | 0.000 | ND6 | Drosophila | melanogaster |
| 0.000 | 0.006 | 0.074 | ND5 | Drosophila | melanogaster |
| 0.000 | 0.008 | 0.000 | COX1 | Drosophila | melanogaster |
| 0.021 | 0.004 | 0.072 | ND1 | Drosophila | melanogaster |
| -1.000 | 0.000 | 0.000 | ATP8 | Drosophila | melanogaster |
| 1.000 | 0.018 | 0.000 | ND4L | Drosophila | melanogaster |
| 0.047 | 0.001 | 0.288 | ND5 | Homo | sapiens |
| 0.682 | 0.004 | 0.693 | ND4L | Homo | sapiens |
| -1.000 | 0.000 | 0.000 | ATP8 | Homo | sapiens |
| 0.690 | 0.001 | 1.228 | COX2 | Homo | sapiens |
| 0.010 | 0.001 | 0.060 | ND4 | Homo | sapiens |
| 1.000 | 0.001 | 0.000 | COX3 | Homo | sapiens |
| 0.043 | 0.002 | 0.102 | ND6 | Homo | sapiens |
| 0.013 | 0.002 | 0.205 | ND1 | Homo | sapiens |
| 0.118 | 0.002 | 0.000 | ATP6 | Homo | sapiens |
| 0.019 | 0.001 | 0.111 | COX1 | Homo | sapiens |
| -1.000 | 0.000 | 0.000 | ND3 | Homo | sapiens |
| 0.761 | 0.001 | 0.647 | ND2 | Homo | sapiens |
| 0.259 | 0.001 | 0.520 | CYTB | Homo | sapiens |
| -1.000 | 0.000 | 0.000 | ATP8 | Strongylocentrotus | intermedius |
| 0.000 | 0.004 | 0.170 | ND5 | Strongylocentrotus | intermedius |
| 0.005 | 0.002 | 0.000 | COX3 | Strongylocentrotus | intermedius |
| 0.000 | 0.006 | 0.000 | COX2 | Strongylocentrotus | intermedius |
| 0.000 | 0.003 | 0.103 | ND4 | Strongylocentrotus | intermedius |
| 0.000 | 0.005 | 0.036 | ATP6 | Strongylocentrotus | intermedius |
| 0.016 | 0.003 | 0.082 | ND1 | Strongylocentrotus | intermedius |
| 0.000 | 0.002 | 0.098 | ND2 | Strongylocentrotus | intermedius |
| 0.000 | 0.002 | 0.000 | COX1 | Strongylocentrotus | intermedius |
| 0.188 | 0.002 | 0.000 | ND4L | Strongylocentrotus | intermedius |
| 0.106 | 0.003 | 0.000 | ND3 | Strongylocentrotus | intermedius |
| 0.006 | 0.002 | 0.000 | ND6 | Strongylocentrotus | intermedius |
| 0.000 | 0.002 | 0.000 | CYTB | Strongylocentrotus | intermedius |

**Table S-6. Nucleotide diversity permutation test results for non-*Beroe* samples** The columns are: *p-val*: The Monte-Carlo p-values that the sequence's $\pi N/\pi S$ values are the result of negative selection rather than $\pi N/\pi S$ from randomly shuffled simulation sequences. A p-value of $-1$ indicates that to value could not be calculated due to a lack of nucleotide diversity. The column *pi* is simply the observed nucleotide diversity ($\pi$). The column *piNpiS* is the observed ratio of nonsynonymous to synonymous diversity ratio, or $\pi N/\pi S$, in the locus. The $\pi$ and $\pi N/\pi S$ values were calculated with biopython's cal_dn_ds function.

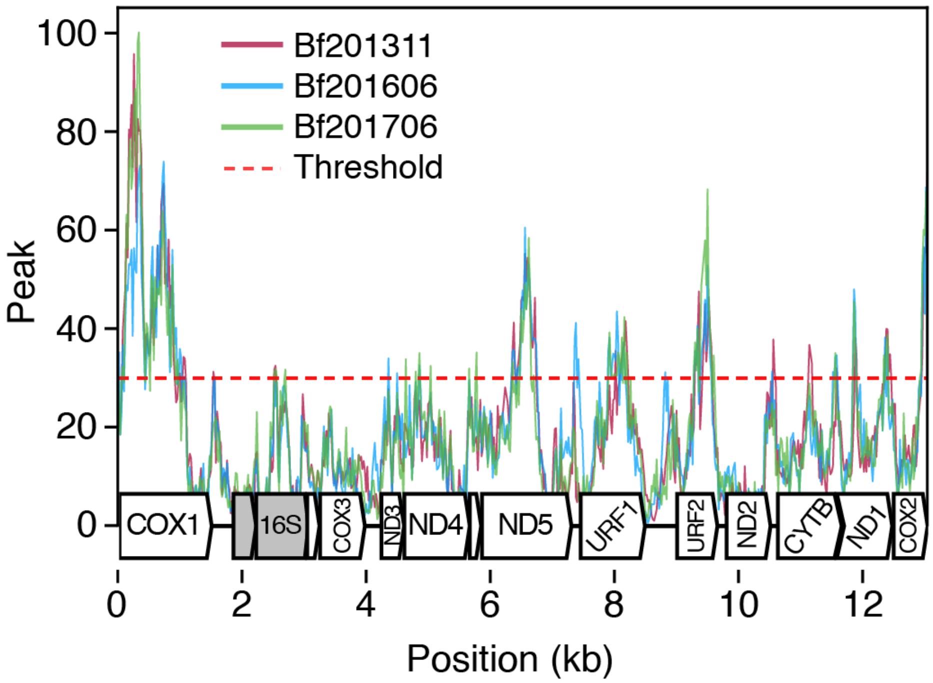


**Figure S-6. Fourier transform analysis.** The Fourier Transform FTG-WINDOW analysis shows strong trinucleotide periodicity that corresponds to protein-coding sequence between 0-1000 bp (COX1), around 6500 bp (ND5), around 7800 bp (URF1), around 9300 bp (URF2). The Y axis indicates increased nucleotide periodicity, a proxy for protein coding likelihood at that locus.

## Samples used in phylogenetic analyses

| | Name | citation | Phylum | Class | Genus | Species |
|---|---|---|---|---|---|---|
| 1 | AF_538053 | (Lang et al., 2002) | Choanozoa | Choanoflagellatea | Monosiga | brevicolis |
| 2 | JN_392469 | (Kohn et al., 2012) | Ctenophora | Tentaculata | Pleurobrachia | bachei |
| 3 | KY_778696 | (Fallon et al., 2018) | Arthropoda | Insecta | Photinus | pyralis |
| 4 | LN_898113 | (Arafat et al., 2018) | Ctenophora | Tentaculata | Coeloplana | loyai |
| 5 | LN_898114 | (Arafat et al., 2018) | Ctenophora | Tentaculata | Coeloplana | yulianicorum |
| 6 | LN_898115 | (Arafat et al., 2018) | Ctenophora | Tentaculata | Vallicula | multiformis |
| 7 | MG_655622 | (this study) | Ctenophora | Nuda | Beroe | forskalii |
| 8 | MG_655623 | (this study) | Ctenophora | Nuda | Beroe | forskalii |
| 9 | MG_655624 | (this study) | Ctenophora | Nuda | Beroe | forskalii |
| 10 | NC_000834 | (Naylor and Brown, 1998) | Chordata | Leptocardii | Branchiostoma | floridae |
| 11 | NC_001453 | (Qureshi and Jacobs, 1993) | Echinodermata | Echinoidea | Strongylocentrotus | purpuratus |
| 12 | NC_001715 | (Paquin and Lang, 1996) | Blastocladiomycota | Blastocladiomycetes | Allomyces | macrogynus |
| 13 | NC_003052 | (Forget et al., 2002) | Chytridiomycota | Chytridiomycetes | Spizellomycetales | punctatus |
| 14 | NC_003053 | (Forget et al., 2002) | Chytridiomycota | Chytridiomycetes | Rhizophydium | graminis |
| 15 | NC_005306 | (Ogoh and Ohmiya, 2004) | Euarthropoda | Ostracoda | Vargula | hilgendorfii |
| 16 | NC_006836 | (Seif et al., 2005) | Zygomycota | Mucoromycotina | Rhizopus | oryzae |
| 17 | NC_006837 | (Seif et al., 2005) | Zygomycota | Kickxellomycotina | Zancudomyces | culisetae |
| 18 | NC_006894 | (Lavrov et al., 2008) | Porifera | Demospongiae | Axinella | corrugata |
| 19 | NC_006990 | (Wang and Lavrov, 2008) | Porifera | Demospongiae | Geodia | neptuni |
| 20 | NC_006991 | (Lavrov et al., 2005) | Porifera | Demospongiae | Tethya | actinia |
| 21 | NC_007893 | (Akasaki et al., 2006) | Mollusca | Cephalopoda | Watasenia | scintillans |
| 22 | NC_008151 | (Dellaporta et al., 2006) | Placozoa | | Trichoplax | adhaerens |
| 23 | NC_008446 | (Shao et al., 2006) | Cnidaria | Scyphozoa | Aurelia | aurita |
| 24 | NC_008556 | (Bourlat et al., 2006) | Xenacoelomorpha | | Xenoturbella | bocki |
| 25 | NC_008832 | (Signorovitch et al., 2007) | Placozoa | | Placozoan | sp |
| 26 | NC_008833 | (Signorovitch et al., 2007) | Placozoa | | Placozoan | sp |
| 27 | NC_008834 | (Signorovitch et al., 2007) | Placozoa | | Placozoan | sp |
| 28 | NC_010201 | (Lavrov et al., 2008) | Porifera | Demospongiae | Amphimedon | compressa |
| 29 | NC_010203 | (Lavrov et al., 2008) | Porifera | Demospongiae | Aplysina | fulva |
| 30 | NC_010207 | (Lavrov et al., 2008) | Porifera | Demospongiae | Iotrochota | birotulata |
| 31 | NC_010208 | (Lavrov et al., 2008) | Porifera | Demospongiae | Chondrilla | sp |
| 32 | NC_010212 | (Lavrov et al., 2008) | Porifera | Demospongiae | Halisarca | dujardini |
| 33 | NC_010216 | (Lavrov et al., 2008) | Porifera | Demospongiae | Igernella | notabilis |
| 34 | NC_010218 | (Lavrov et al., 2008) | Porifera | Demospongiae | Vaceletia | GW948 |
| 35 | NC_010496 | (Lukić-Bilela et al., 2008) | Porifera | Demospongiae | Suberites | domuncula |
| 36 | NC_011360 | (Tambor et al., 2008) | Blastocladiomycota | Blastocladiomycetes | Blastocladiella | emersonii |
| 37 | NC_012761 | (Matsui et al., 2009) | Chordata | Mammalia | Galago | senegalensis |
| 38 | NC_013662 | (Erpenbeck et al., 2009) | Porifera | Demospongiae | Ircinia | strobilina |
| 39 | NC_014856 | (Gazave et al., 2010) | Porifera | Homoscleromorpha | Oscarella | viridis |
| 40 | NC_014860 | (Gazave et al., 2010) | Porifera | Homoscleromorpha | Plakina | jani |
| 41 | NC_014884 | (Gazave et al., 2010) | Porifera | Homoscleromorpha | Plakina | monolopha |
| 42 | NC_014888 | (Gazave et al., 2010) | Porifera | Homoscleromorpha | Oscarella | tuberculata |
| 43 | NC_015309 | NA | Placozoa | | Placozoan | sp |
| 44 | NC_016117 | (Pett et al., 2011) | Ctenophora | Tentaculata | Mnemiopsis | leidyi |
| 45 | NC_016431 | NA | Porifera | Demospongiae | Eunapius | subterraneus |
| 46 | NC_016466 | (Kayal et al., 2012) | Cnidaria | Scyphozoa | Cassiopea | frondosa |
| 47 | NC_016467 | (Kayal et al., 2012) | Cnidaria | Hydrozoa | Cubaia | aphrodite |
| 48 | NC_018378 | NA | Cnidaria | Anthozoa | Renilla | muelleri |
| 49 | NC_018537 | (Zou et al., 2012) | Cnidaria | Hydrozoa | Craspedacusta | sowerbyi |
| 50 | NC_020030 | NA | Priapulida | Priapulimorpha | Halicryptus | spinulosus |
| 51 | NC_020459 | (Park et al., 2012) | Cnidaria | Scyphozoa | Chrysaora | quinquecirrha |
| 52 | NC_021406 | (Pan et al., 2014) | Cnidaria | Hydrozoa | Hydra | sinensis |
| 53 | NC_023834 | (del Cerro et al., 2016) | Porifera | Demospongiae | Polymastia | littoralis |
| 54 | NC_027419 | (Jourda et al., 2015) | Porifera | Hexactinellida | Oopsacas | minuta |
| 55 | NC_028054 | (Haen et al., 2014) | Porifera | Hexactinellida | Vazella | pourtalesi |
| 56 | NC_030337 | (Li et al., 2016) | Cnidaria | Staurozoa | Haliclystus | antarcticus |
| 57 | NC_031810 | (Chen et al., 2016) | Chordata | Chondrichthyes | Etmopterus | pusillus |
| 58 | NC_034783 | NA | Annelida | Clitellata | Pontoscolex | corethrurus |
| 59 | NC_035665 | (Poliseno et al., 2017) | Cnidaria | Anthozoa | Eugorgia | mutabilis |
| 60 | NC_035667 | (Poliseno et al., 2017) | Cnidaria | Anthozoa | Eunicella | cavolinii |
| 61 | NC_035740 | (Wang and Sun, 2017) | Cnidaria | Scyphozoa | Nemopilema | nomurai |
| 62 | NC_040151 | (Galaska et al., 2019) | Echinodermata | Ophiuroidea | Ophiosteira | antarctica |

**Table S-7.** Table of samples included in the phylogenetic analysis.

## Phylogenetic trees - individual

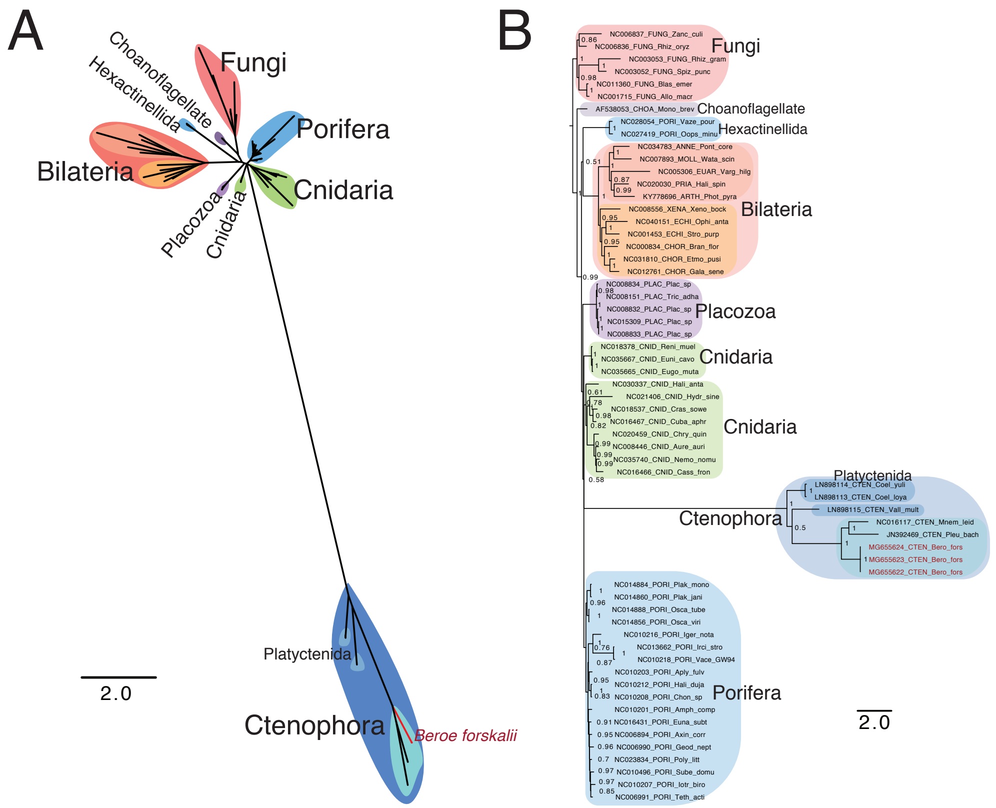


**Figure S-7. Phylobayes without using Guidance2**. An amino acid tree constructed with the COX1, COX2, COX3, CYTB, ND1, ND3, and ND5 genes. Guidance2 (Sela et al., 2015) **was not** used to remove unreliable alignment regions. This tree was constructed in Phylobayes using the CAT + GTR + Γ model by running three chains for approximately 10250 iterations. The consensus tree was generated by using the first 2560 trees as burn-in, sampling every 10 subsequent 10 trees. The max difference was 0.088, indicating proper convergence between the three chains (See Table S-8.)

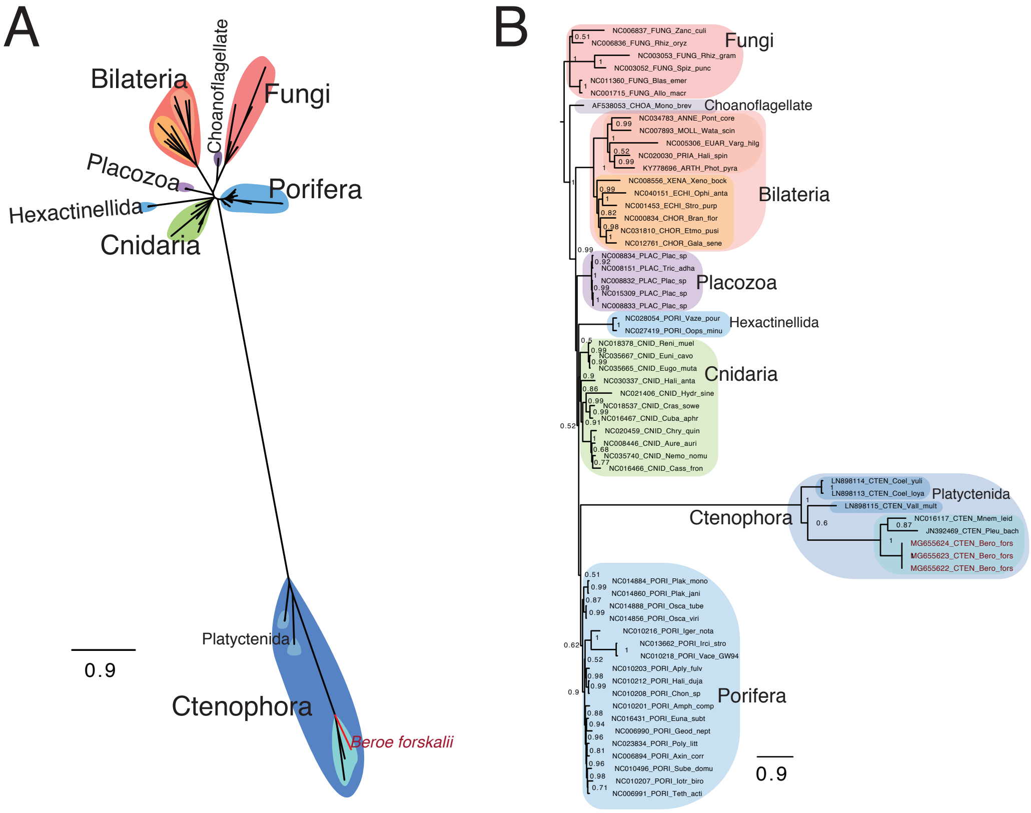


**Figure S-8. Phylobayes using Guidance2** An amino acid tree constructed with the COX1, COX2, COX3, CYTB, ND1, ND3, and ND5 genes. Guidance2 (Sela et al., 2015) was used to remove unreliable alignment regions. This tree was constructed in Phylobayes using the CAT + GTR + Γ model by running three chains for approximately 34500 iterations. The consensus tree was generated by using the first 8645 trees as burn-in, sampling every 10 subsequent 10 trees. The max difference was 0.0849, indicating proper convergence between the three chains (See Table S-8.)

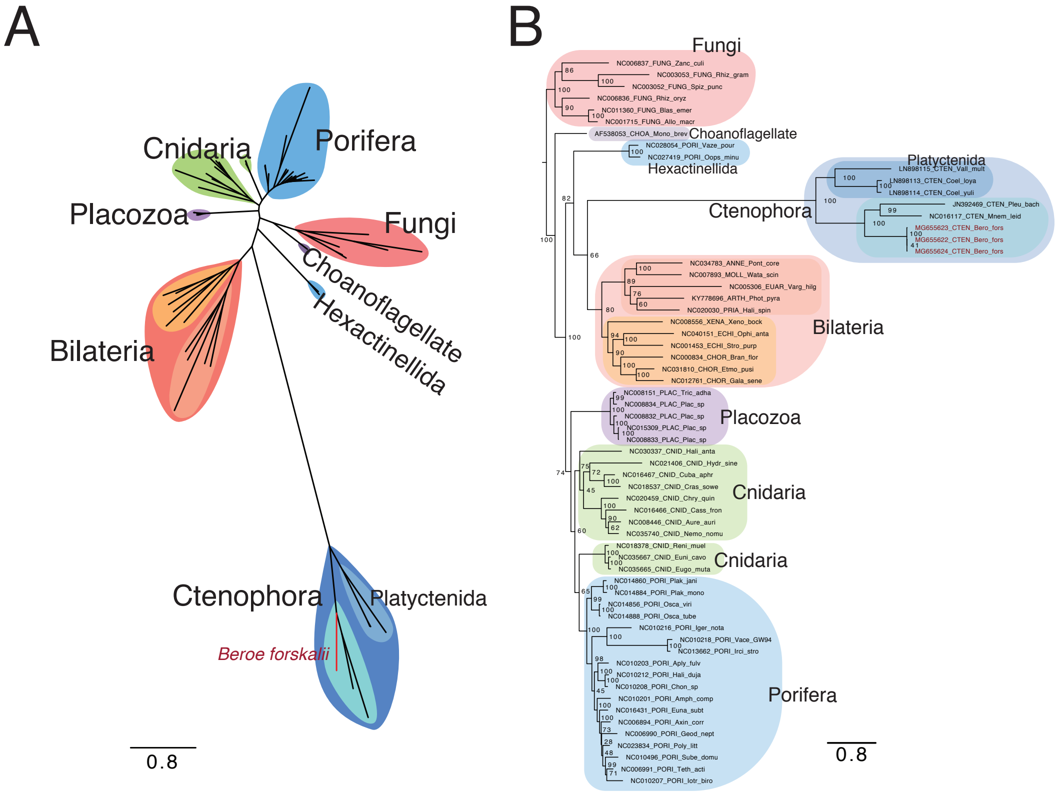


**Figure S-9. RAxML without using Guidance2** An amino acid tree constructed with the COX1, COX2, COX3, CYTB, ND1, ND3, and ND5 genes. Guidance2 (Sela et al., 2015) **was not** used to remove unreliable alignment regions. This tree was constructed in RAxML using the PROTCATWAG model with a single partition for all loci and rapid bootstrapping.

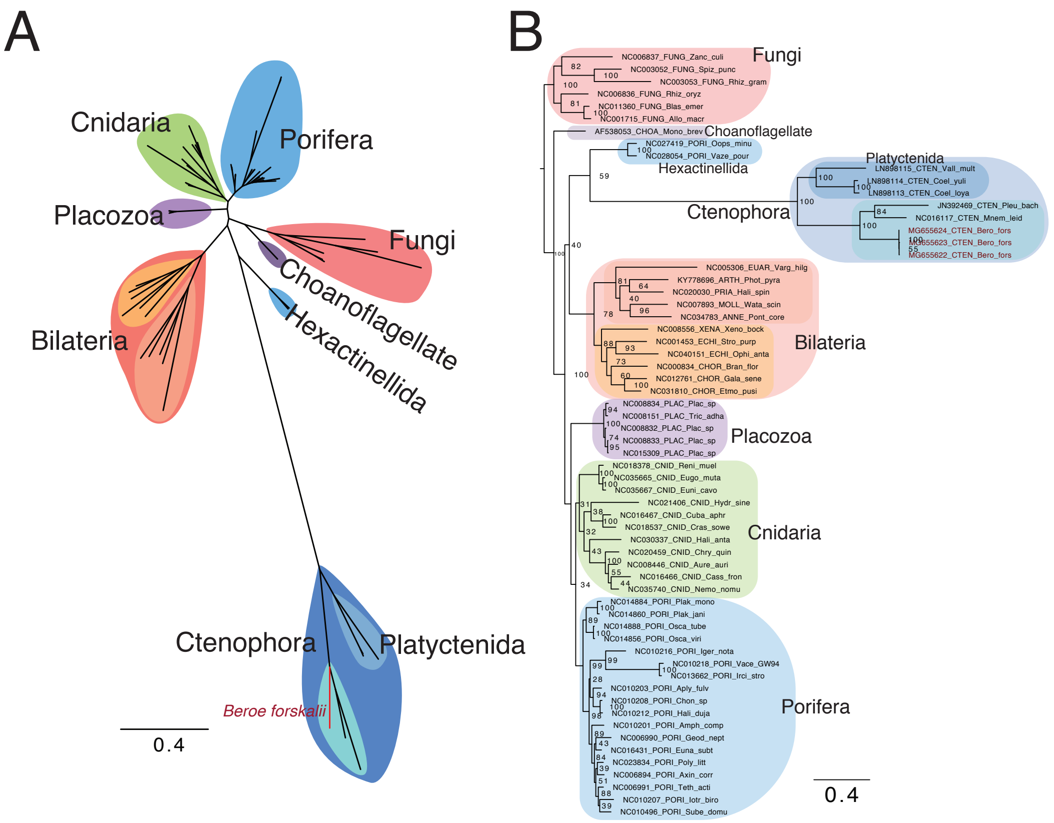


**Figure S-10. RAxML using Guidance2** An amino acid tree constructed with the COX1, COX2, COX3, CYTB, ND1, ND3, and ND5 genes. Guidance2 (Sela et al., 2015) was not used to remove unreliable alignment regions. This tree was constructed in RAxML using the PROTCATWAG model with a single partition for all loci and rapid bootstrapping.

## Phylogenetic trees composite figure

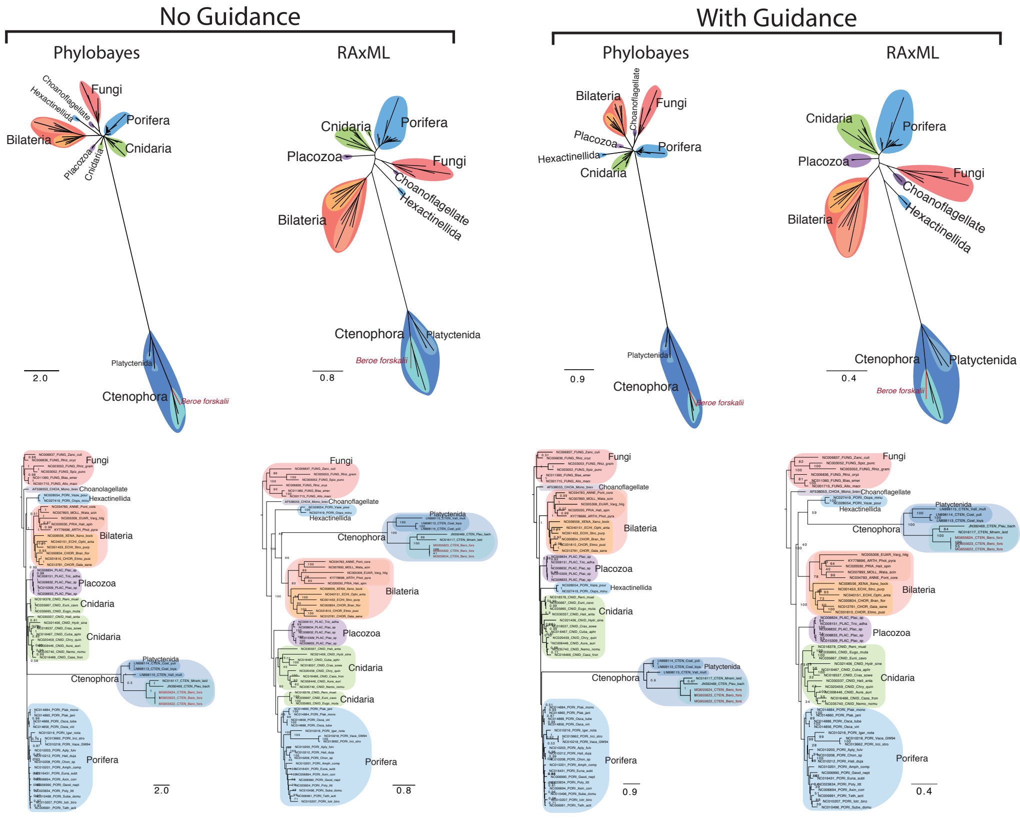


**Figure S-11. All Phylogenetic Trees.** This plot is Figures S-7, S-8, S-9, S-10 side-by-side for comparison.

## Statistics of Phylobayes Trees

| | | | | | number of iterations | | | number of trees | | | | | |
|---|---|---|---|---|---|---|---|---|---|---|---|---|---|
| loci | OG | G | Burnin | S | chain1 | chain2 | chain3 | chain1 | chain2 | chain3 | maxdiff | meandiff | diff |
| limited | yes | no | 2560 | 10 | 10243 | 10277 | 10290 | 768 | 771 | 773 | 0.0880 | 0.0061 | 0.0095 |
| limited | yes | yes | 8645 | 10 | 34581 | 34478 | 34590 | 2593 | 2583 | 2594 | 0.0849 | 0.0059 | 0.0062 |
| complete | no | no | 1306 | 10 | 5224 | 5199 | 5131 | 391 | 389 | 382 | 0.0580 | 0.0027 | 0.0481 |

**Table S-8.** Phylobayes convergence results.

| | | | loglik | | length | | alpha | | Nmode | |
|---|---|---|---|---|---|---|---|---|---|---|
| loci | OG | G | eff_size | diff | eff_size | diff | eff_size | diff | eff_size | diff |
| limited | yes | no | 315 | 0.0399 | 165 | 0.2720 | 478 | 0.0999 | 542 | 0.1080 |
| limited | yes | yes | 1443 | 0.0952 | 4414 | 0.0452 | 8583 | 0.0233 | 1830 | 0.0873 |
| complete | no | no | 158 | 0.2027 | 118 | 0.1955 | 268 | 0.1344 | 260 | 0.1283 |

**Table S-9.** Phylobayes convergence results, part 2.

| | | | statent | | statalpha | | rrent | | rrmean | |
|---|---|---|---|---|---|---|---|---|---|---|
| loci | OG | G | eff_size | diff | eff_size | diff | eff_size | diff | eff_size | diff |
| limited | yes | no | 2998 | 0.0954 | 1262 | 0.1216 | 379 | 0.2206 | 7684 | 0.0096 |
| limited | yes | yes | 3819 | 0.0622 | 2645 | 0.0213 | 4195 | 0.0186 | 25834 | 0.0062 |
| complete | no | no | 230 | 0.1997 | 833 | 0.1385 | 617 | 0.0311 | 3163 | 0.0481 |

**Table S-10.** Phylobayes convergence results, part 3.

## Tables pertaining to nucleotide diversity in *B. forskalii* genes

| | Number of N or S sites of type: | | | | | | | |
|---|---|---|---|---|---|---|---|---|
| | MM | | IM | | MM + IM | | TM | |
| | N | S | N | S | N | S | N | S |
| **COX1** | 3 | 9 | 0 | 8 | 3 | 17 | 0 | 44 |
| **COX2** | 0 | 2 | 0 | 2 | 0 | 4 | 0 | 4 |
| **COX3** | 0 | 0 | 1 | 3 | 1 | 3 | 0 | 4 |
| **CYTB** | 0 | 4 | 0 | 8 | 0 | 12 | 2 | 26 |
| **ND1** | 0 | 3 | 0 | 7 | 0 | 10 | 1 | 9 |
| **ND2** | 1 | 3 | 0 | 3 | 1 | 6 | 1 | 15 |
| **ND3** | 0 | 0 | 1 | 0 | 1 | 0 | 0 | 7 |
| **ND4** | 0 | 5 | 1 | 2 | 1 | 7 | 3 | 17 |
| **ND4L** | 0 | 0 | 0 | 0 | 0 | 0 | 2 | 1 |
| **ND5** | 1 | 2 | 1 | 4 | 2 | 6 | 3 | 16 |
| **ND6** | 0 | 0 | 0 | 1 | 0 | 1 | 1 | 4 |
| **URF1** | 3 | 1 | 4 | 3 | 7 | 4 | 8 | 6 |
| **URF2** | 5 | 2 | 5 | 13 | 10 | 15 | 17 | 16 |

**Table S-11.** Number of sites that have either predominantly synonymous mutations or predominantly nonsynonymous mutations. Sites are either N, S, or contain no mutations. There are no sites that are double counted as both N and S, for example. Abbreviations: MM = Mitochondrial Matrix, IM = Intermembrane space, TM = Transmembrane, S = Synonymous site, N = Nonsynonymous site.

| | Number of sites of type: | | | | |
|---|---|---|---|---|---|
| | MM | IM | MM + IM | TM | MM + IM + TM |
| **COX1** | 99 | 114 | 213 | 293 | 506 |
| **COX2** | 70 | 47 | 117 | 69 | 186 |
| **COX3** | 23 | 66 | 89 | 157 | 246 |
| **CYTB** | 40 | 63 | 103 | 247 | 350 |
| **ND1** | 20 | 72 | 92 | 211 | 303 |
| **ND2** | 20 | 40 | 60 | 181 | 241 |
| **ND3** | 13 | 18 | 31 | 92 | 123 |
| **ND4** | 52 | 43 | 95 | 264 | 359 |
| **ND4L** | 6 | 3 | 9 | 46 | 55 |
| **ND5** | 73 | 62 | 135 | 356 | 491 |
| **ND6** | 6 | 14 | 20 | 46 | 66 |
| **URF1** | 61 | 111 | 172 | 181 | 353 |
| **URF2** | 23 | 60 | 83 | 138 | 221 |

**Table S-12.** Number of sites for each protein domain type. Sites are either N, S, or contain no mutations. There are no sites that are double counted as both N and S, for example. Abbreviations: MM = Mitochondrial Matrix, IM = Intermembrane space, TM = Transmembrane, S = Synonymous site, N = Nonsynonymous site.

| | In the IM, MM, or TM sites, what percent are N or S? | | | | | | | |
|---|---|---|---|---|---|---|---|---|
| | IM | | MM | | IM or MM | | TM | |
| | N | S | N | S | N | S | N | S |
| **COX1** | 0.00% | 7.02% | 3.03% | 9.09% | 1.41% | 7.98% | 0.00% | 15.02% |
| **COX2** | 0.00% | 4.26% | 0.00% | 2.86% | 0.00% | 3.42% | 0.00% | 5.80% |
| **COX3** | 1.52% | 4.55% | 0.00% | 0.00% | 1.12% | 3.37% | 0.00% | 2.55% |
| **CYTB** | 0.00% | 12.70% | 0.00% | 10.00% | 0.00% | 11.65% | 0.81% | 10.53% |
| **ND1** | 0.00% | 9.72% | 0.00% | 15.00% | 0.00% | 10.87% | 0.47% | 4.27% |
| **ND2** | 0.00% | 7.50% | 5.00% | 15.00% | 1.67% | 10.00% | 0.55% | 8.29% |
| **ND3** | 5.56% | 0.00% | 0.00% | 0.00% | 3.23% | 0.00% | 0.00% | 7.61% |
| **ND4** | 2.33% | 4.65% | 0.00% | 9.62% | 1.05% | 7.37% | 1.14% | 6.44% |
| **ND4L** | 0.00% | 0.00% | 0.00% | 0.00% | 0.00% | 0.00% | 4.35% | 2.17% |
| **ND5** | 1.61% | 6.45% | 1.37% | 2.74% | 1.48% | 4.44% | 0.84% | 4.49% |
| **ND6** | 0.00% | 7.14% | 0.00% | 0.00% | 0.00% | 5.00% | 2.17% | 8.70% |
| **URF1** | 3.60% | 2.70% | 4.92% | 1.64% | 4.07% | 2.33% | 4.42% | 3.31% |
| **URF2** | 8.33% | 21.67% | 21.74% | 8.70% | 12.05% | 18.07% | 12.32% | 11.59% |

**Table S-13.** The percent of each domain type in each locus that is predominantly N or S types. Sites are either N, S, or contain no mutations. There are no sites that are double counted as both N and S, for example. Abbreviations: MM = Mitochondrial Matrix, IM = Intermembrane space, TM = Transmembrane, S = Synonymous site, N = Nonsynonymous site.

| | What percent of all sites in the locus are IM N, IM S, MM N, MM S, et cetera? | | | | | | | |
|---|---|---|---|---|---|---|---|---|
| | IM | | MM | | IM or MM | | TM | |
| | N | S | N | S | N | S | N | S |
| **COX1** | 0.00% | 1.58% | 0.59% | 1.78% | 0.59% | 3.36% | 0.59% | 3.36% |
| **COX2** | 0.00% | 1.08% | 0.00% | 1.08% | 0.00% | 2.15% | 0.00% | 2.15% |
| **COX3** | 0.41% | 1.22% | 0.00% | 0.00% | 0.41% | 1.22% | 0.41% | 1.22% |
| **CYTB** | 0.00% | 2.29% | 0.00% | 1.14% | 0.00% | 3.43% | 0.00% | 3.43% |
| **ND1** | 0.00% | 2.31% | 0.00% | 0.99% | 0.00% | 3.30% | 0.00% | 3.30% |
| **ND2** | 0.00% | 1.24% | 0.41% | 1.24% | 0.41% | 2.49% | 0.41% | 2.49% |
| **ND3** | 0.81% | 0.00% | 0.00% | 0.00% | 0.81% | 0.00% | 0.81% | 0.00% |
| **ND4** | 0.28% | 0.56% | 0.00% | 1.39% | 0.28% | 1.95% | 0.28% | 1.95% |
| **ND4L** | 0.00% | 0.00% | 0.00% | 0.00% | 0.00% | 0.00% | 0.00% | 0.00% |
| **ND5** | 0.20% | 0.81% | 0.20% | 0.41% | 0.41% | 1.22% | 0.41% | 1.22% |
| **ND6** | 0.00% | 1.52% | 0.00% | 0.00% | 0.00% | 1.52% | 0.00% | 1.52% |
| **URF1** | 1.13% | 0.85% | 0.85% | 0.28% | 1.98% | 1.13% | 1.98% | 1.13% |
| **URF2** | 2.26% | 5.88% | 2.26% | 0.90% | 4.52% | 6.79% | 4.52% | 6.79% |

**Table S-14.** The percent of all sites in each gene that are N or S types. Sites are either N, S, or contain no mutations. There are no sites that are double counted as both N and S, for example. Abbreviations: MM = Mitochondrial Matrix, IM = Intermembrane space, TM = Transmembrane, S = Synonymous site, N = Nonsynonymous site.

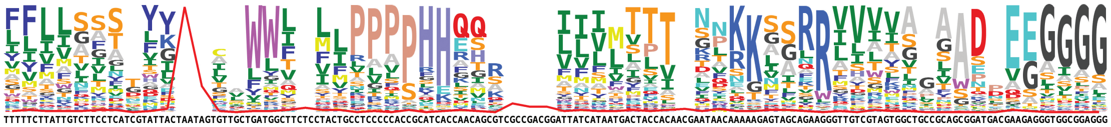

**Figure S-12.** FACIL genetic code logo. There are no codons that are predicted to have a reassignment relative to the Mold, Protozoan, and Coelenterate mitochondrial code. The top amino acid in the stack was the most frequent in the alignment.

| codon | FACIL | stpRF12 | AAokRF3 | top_AA | standard |
|---|---|---|---|---|---|
| TTT | F | 0.01 | 0.96 | F | F |
| TTC | F | 0.02 | 0.99 | F | F |
| TTA | L | 0.01 | 0.97 | L | L |
| TTG | L | 0.01 | 0.97 | L | L |
| TCT | S | 0.01 | 0.92 | S | S |
| TCC | S | 0.02 | 0.89 | S | S |
| TCA | S | 0.02 | 0.96 | S | S |
| TCG | X | 0 | 0.31 | T | S |
| TAT | Y | 0.01 | 0.99 | Y | Y |
| TAC | Y | 0.03 | 0.93 | Y | Y |
| TAA | * | 0.97 | n.a. | * | * |
| TAG | X | 0.24 | n.a. | * | * |
| TGT | C | 0.01 | 0.58 | C | C |
| TGC | X | 0 | 0.22 | A | C |
| TGA | W | 0.01 | 1 | W | W |
| TGG | W | 0.01 | 0.99 | W | W |
| CTT | L | 0.02 | 0.96 | L | L |
| CTC | X | 0.04 | n.a. | * | L |
| CTA | L | 0.02 | 0.97 | L | L |
| CTG | L | 0.02 | 0.77 | L | L |
| CCT | P | 0 | 1 | P | P |
| CCC | P | 0.01 | 0.97 | P | P |
| CCA | P | 0 | 1 | P | P |
| CCG | P | 0.02 | 0.91 | P | P |
| CAT | H | 0 | 0.99 | H | H |
| CAC | H | 0.01 | 0.98 | H | H |
| CAA | Q | 0.02 | 0.91 | Q | Q |
| CAG | Q | 0.01 | 0.88 | Q | Q |
| CGT | X | 0 | 0.45 | R | R |
| CGC | X | 0.08 | n.a. | * | R |
| CGA | X | 0.05 | n.a. | * | R |
| CGG | X | 0.05 | n.a. | * | R |
| ATT | I | 0.01 | 0.94 | I | I |
| ATC | I | 0.02 | 0.9 | I | I |
| ATA | I | 0.01 | 0.94 | I | I |
| ATG | M | 0.01 | 0.79 | M | M |
| ACT | T | 0.02 | 0.97 | T | T |
| ACC | T | 0.01 | 0.97 | T | T |
| ACA | T | 0.02 | 0.93 | T | T |
| ACG | X | 0.03 | n.a. | * | T |
| AAT | N | 0.01 | 0.96 | N | N |
| AAC | N | 0.03 | 0.83 | N | N |
| AAA | K | 0.01 | 0.99 | K | K |
| AAG | K | 0.01 | 0.91 | K | K |
| AGT | S | 0.02 | 0.89 | S | S |
| AGC | S | 0.02 | 0.84 | S | S |
| AGA | R | 0.01 | 0.99 | R | R |
| AGG | R | 0 | 0.96 | R | R |
| GTT | V | 0.01 | 0.98 | V | V |
| GTC | V | 0 | 0.99 | V | V |
| GTA | V | 0.02 | 0.97 | V | V |
| GTG | V | 0.01 | 0.93 | V | V |
| GCT | A | 0.01 | 0.98 | A | A |
| GCC | X | 0 | 0.32 | G | A |
| GCA | A | 0 | 0.97 | A | A |
| GCG | A | 0 | 0.93 | A | A |
| GAT | D | 0 | 0.96 | D | D |
| GAC | X | 0 | 0.25 | P | D |
| GAA | E | 0.02 | 1 | E | E |
| GAG | E | 0.01 | 0.93 | E | E |
| GGT | G | 0 | 1 | G | G |
| GGC | G | 0 | 0.9 | G | G |
| GGA | G | 0 | 1 | G | G |
| GGG | G | 0 | 1 | G | G |

**Table S-15.** Results of the Facil analysis. Columns are the codons, the FACIL amino acid code (X means unable to determine), stpRF12 and AAokRF3 are probability scores from the random forest classifiers, top_AA is the most frequent amino acid from the six translation frame alignments, and standard is the amino acid in the standard code for that codon.

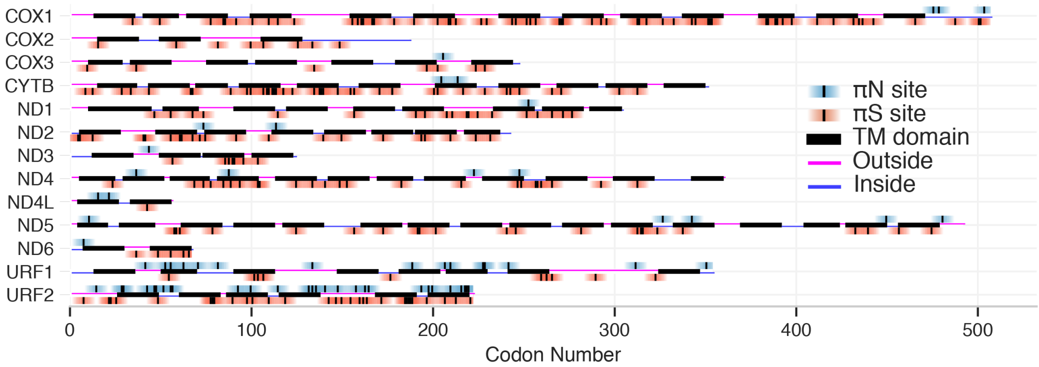


**Figure S-13. Heterogeneity of Nucleotide Diversity in mitochondrial genes.** Each row represents a single gene with marked positions of nonsynonymous or synonymous substitution positions. Each gene was structurally annotated with TMHMM: horizontal pink lines are protein regions exposed to the mitochondrial inter membrane space, horizontal blue lines are protein regions exposed to the mitochondrial matrix, and horizontal black lines are transmembrane domains. AA positions with at least one nonsynonymous site are marked as N sites above each sequence. AA positions with at least one synonymous substitution are marked as *S sites* below each sequence. The actual position of the substitution in the *N* and *S* sites is the vertical black bar, while the gradient highlighting is simply a visualization aid.

## REFERENCES


Akasaki, T., Nikaido, M., Tsuchiya, K., Segawa, S., Hasegawa, M., and Okada, N. (2006). Extensive mitochondrial gene arrangements in coleoid Cephalopoda and their phylogenetic implications. *Mol. Phylogenet. Evol.*, 38(3):648–658.

Arafat, H., Alamaru, A., Gissi, C., and Huchon, D. (2018). Extensive mitochondrial gene rearrangements in ctenophora: insights from benthic platyctenida. *BMC Evol. Biol.*, 18(1):65.

Bourlat, S. J., Juliusdottir, T., Lowe, C. J., Freeman, R., Aronowicz, J., Kirschner, M., Lander, E. S., Thorndyke, M., Nakano, H., Kohn, A. B., Heyland, A., Moroz, L. L., Copley, R. R., and Telford, M. J. (2006). Deuterostome phylogeny reveals monophyletic chordates and the new phylum Xenoturbellida. *Nature*, 444(7115):85–88.

Chen, H., Chen, X., Gu, X., Wan, H., Chen, X., and Ai, W. (2016). The phylogenomic position of the smooth lanternshark *Etmopterus pusillus* (Squaliformes: Etmopteridae) inferred from the mitochondrial genome. *Mitochondrial DNA Part B*, 1(1):341–342.

Cock, P. J. A., Antao, T., Chang, J. T., Chapman, B. A., Cox, C. J., Dalke, A., Friedberg, I., Hamelryck, T., Kauff, F., Wilczynski, B., and de Hoon, M. J. L. (2009). Biopython: freely available python tools for computational molecular biology and bioinformatics. *Bioinformatics*, 25(11):1422–1423.

del Cerro, C., Peñalver, A., Cuevas, C., de la Calle, F., Galán, B., and García, J. L. (2016). Complete mitochondrial genome of *Polymastia littoralis* (Demospongiae, Polymastiidae). *Mitochondrial DNA A DNA Mapp Seq Anal*, 27(1):312–313.

Dellaporta, S. L., Xu, A., Sagasser, S., Jakob, W., Moreno, M. A., Buss, L. W., and Schierwater, B. (2006). Mitochondrial genome of *Trichoplax adhaerens* supports placozoa as the basal lower metazoan phylum. *Proc. Natl. Acad. Sci. U. S. A.*, 103(23):8751–8756.

Erpenbeck, D., Voigt, O., Wörheide, G., and Lavrov, D. V. (2009). The mitochondrial genomes of sponges provide evidence for multiple invasions by repetitive hairpin-forming elements (RHE). *BMC Genomics*, 10:591.

Fallon, T. R., Lower, S. E., Chang, C.-H., Bessho-Uehara, M., Martin, G. J., Bewick, A. J., Behringer, M., Debat, H. J., Wong, I., Day, J. C., Suvorov, A., Silva, C. J., Stanger-Hall, K. F., Hall, D. W., Schmitz, R. J., Nelson, D. R., Lewis, S. M., Shigenobu, S., Bybee, S. M., Larracuente, A. M., Oba, Y., and Weng, J.-K. (2018). Firefly genomes illuminate parallel origins of bioluminescence in beetles. *Elife*, 7:e36495.

Forget, L., Ustinova, J., Wang, Z., Huss, V. A. R., and Lang, B. F. (2002). *Hyaloraphidium curvatum*: a linear mitochondrial genome, tRNA editing, and an evolutionary link to lower fungi. *Mol. Biol. Evol.*, 19(3):310–319.

Galaska, M. P., Li, Y., Kocot, K. M., Mahon, A. R., and Halanych, K. M. (2019). Conservation of mitochondrial genome arrangements in brittle stars (Echinodermata, Ophiuroidea). *Mol. Phylogenet. Evol.*, 130:115–120.

Gazave, E., Lapébie, P., Renard, E., Vacelet, J., Rocher, C., Ereskovsky, A. V., Lavrov, D. V., and Borchiellini, C. (2010). Molecular phylogeny restores the supra-generic subdivision of homoscleromorph sponges (Porifera, Homoscleromorpha). *PLoS One*, 5(12):e14290.

Guillet, V., Chevrollier, A., Cassereau, J., Letournel, F., Gueguen, N., Richard, L., Desquiret, V., Verny, C., Procaccio, V., Amati-Bonneau, P., Reynier, P., and Bonneau, D. (2010). Ethambutol-induced optic neuropathy linked to OPA1 mutation and mitochondrial toxicity. *Mitochondrion*, 10(2):115–124.

Haen, K. M., Pett, W., and Lavrov, D. V. (2014). Eight new mtDNA sequences of glass sponges reveal an extensive usage of +1 frameshifting in mitochondrial translation. *Gene*, 535(2):336–344.

Jourda, C., Santini, S., Rocher, C., Le Bivic, A., and Claverie, J.-M. (2015). Mitochondrial Genome Sequence of the Glass Sponge *Oopsacas minuta*. *Genome Announc.*, 3(4).

Kayal, E., Bentlage, B., Collins, A. G., Kayal, M., Pirro, S., and Lavrov, D. V. (2012). Evolution of linear mitochondrial genomes in medusozoan cnidarians. *Genome Biol. Evol.*, 4(1):1–12.

Kober, K. M. and Bernardi, G. (2013). Phylogenomics of strongylocentrotid sea urchins. *BMC Evol. Biol.*, 13:88.

Kohn, A. B., Citarella, M. R., Kocot, K. M., Bobkova, Y. V., Halanych, K. M., and Moroz, L. L. (2012). Rapid evolution of the compact and unusual mitochondrial genome in the ctenophore,

*Pleurobrachia bachei*. *Mol. Phylogenet. Evol.*, 63(1):203–207.

Lang, B. F., O'Kelly, C., Nerad, T., Gray, M. W., and Burger, G. (2002). The closest unicellular relatives of animals. *Curr. Biol.*, 12(20):1773–1778.

Lavrov, D. V., Forget, L., Kelly, M., and Lang, B. F. (2005). Mitochondrial genomes of two demosponges provide insights into an early stage of animal evolution. *Mol. Biol. Evol.*, 22(5):1231–1239.

Lavrov, D. V., Wang, X., and Kelly, M. (2008). Reconstructing ordinal relationships in the Demospongiae using mitochondrial genomic data. *Mol. Phylogenet. Evol.*, 49(1):111–124.

Li, H. H., Sung, P. J., and Ho, H. C. (2016). The complete mitochondrial genome of the antarctic stalked jellyfish, *Haliclystus antarcticus* Pfeffer, 1889 (Staurozoa: Stauromedusae). *Genome Data*, 8:113–114.

Lukić-Bilela, L., Brandt, D., Pojskić, N., Wiens, M., Gamulin, V., and Müller, W. E. G. (2008). Mitochondrial genome of *Suberites domuncula*: palindromes and inverted repeats are abundant in non-coding regions. *Gene*, 412(1-2):1–11.

Matsui, A., Rakotondraparany, F., Munechika, I., Hasegawa, M., and Horai, S. (2009). Molecular phylogeny and evolution of prosimians based on complete sequences of mitochondrial DNAs. *Gene*, 441(1-2):53–66.

McGuffin, L. J., Bryson, K., and Jones, D. T. (2000). The PSIPRED protein structure prediction server. *Bioinformatics*, 16(4):404–405.

Naylor, G. J. and Brown, W. M. (1998). *Amphioxus* mitochondrial DNA, chordate phylogeny, and the limits of inference based on comparisons of sequences. *Syst. Biol.*, 47(1):61–76.

Nei, M. and Gojobori, T. (1986). Simple methods for estimating the numbers of synonymous and nonsynonymous nucleotide substitutions. *Mol. Biol. Evol.*, 3(5):418–426.

North, B. V., Curtis, D., and Sham, P. C. (2002). A note on the calculation of empirical P values from Monte Carlo procedures. *Am. J. Hum. Genet.*, 71(2):439–441.

Ogoh, K. and Ohmiya, Y. (2004). Complete mitochondrial DNA sequence of the sea-firefly, *Vargula hilgendorfii* (Crustacea, Ostracoda) with duplicate control regions. *Gene*, 327(1):131–139.

Pan, H. C., Qian, X. C., Li, P., Li, X. F., and Wang, A. T. (2014). The complete mitochondrial genome of Chinese green hydra, *Hydra sinensis* (Hydroida: Hydridae). *Mitochondrial DNA*, 25(1):44–45.

Paquin, B. and Lang, B. F. (1996). The mitochondrial DNA of *Allomyces macrogynus*: the complete genomic sequence from an ancestral fungus. *J. Mol. Biol.*, 255(5):688–701.

Park, E., Hwang, D. S., Lee, J. S., Song, J. I., Seo, T. K., and Won, Y. J. (2012). Estimation of divergence times in cnidarian evolution based on mitochondrial protein-coding genes and the fossil record. *Mol. Phylogenet. Evol.*, 62(1):329–345.

Pett, W., Ryan, J. F., Pang, K., Mullikin, J. C., Martindale, M. Q., Baxevanis, A. D., and Lavrov, D. V. (2011). Extreme mitochondrial evolution in the ctenophore *Mnemiopsis leidyi*: Insight from mtDNA and the nuclear genome. *Mitochondrial DNA*, 22(4):130–142.

Poliseno, A., Feregrino, C., Sartoretto, S., Aurelle, D., Wörheide, G., McFadden, C. S., and Vargas, S. (2017). Comparative mitogenomics, phylogeny and evolutionary history of *Leptogorgia* (Gorgoniidae). *Mol. Phylogenet. Evol.*, 115:181–189.

Qureshi, S. A. and Jacobs, H. T. (1993). Two distinct, sequence-specific DNA-binding proteins interact independently with the major replication pause region of sea urchin mtDNA. *Nucleic Acids Res.*, 21(12):2801–2808.

Rani, D. S., Dhandapany, P. S., Nallari, P., Govindaraj, P., Singh, L., and Thangaraj, K. (2010). Mitochondrial DNA 'r' is associated with Noonan syndrome of south India. *Mitochondrion*, 10(2):166–173.

Seif, E., Leigh, J., Liu, Y., Roewer, I., Forget, L., and Lang, B. F. (2005). Comparative mitochondrial genomics in zygomycetes: bacteria-like RNase P RNAs, mobile elements and a close source of the group I intron invasion in angiosperms. *Nucleic Acids Res.*, 33(2):734–744.

Sela, I., Ashkenazy, H., Katoh, K., and Pupko, T. (2015). GUIDANCE2: accurate detection of unreliable alignment regions accounting for the uncertainty of multiple parameters. *Nucleic Acids Res.*, 43(W1):W7–14.

Shao, Z., Graf, S., Chaga, O. Y., and Lavrov, D. V. (2006). Mitochondrial genome of the

moon jelly *Aurelia aurita* (Cnidaria, Scyphozoa): A linear DNA molecule encoding a putative DNA-dependent DNA polymerase. *Gene*, 381:92–101.

Signorovitch, A. Y., Buss, L. W., and Dellaporta, S. L. (2007). Comparative genomics of large mitochondria in placozoans. *PLoS Genet.*, 3(1):e13.

Smith, D. R. and Lee, R. W. (2008). Nucleotide diversity in the mitochondrial and nuclear compartments of *Chlamydomonas reinhardtii*: investigating the origins of genome architecture. *BMC Evol. Biol.*, 8:156.

Tambor, J. H. M., Ribichich, K. F., and Gomes, S. L. (2008). The mitochondrial view of *Blastocladiella emersonii*. *Gene*, 424(1-2):33–39.

van de Loosdrecht, M., Bouzouggar, A., Humphrey, L., Posth, C., Barton, N., Aximu-Petri, A., Nickel, B., Nagel, S., Talbi, E. H., El Hajraoui, M. A., Amzazi, S., Hublin, J. J., Pääbo, S., Schiffels, S., Meyer, M., Haak, W., Jeong, C., and Krause, J. (2018). Pleistocene North African genomes link near eastern and sub-Saharan African human populations. *Science*, 360(6388):548–552.

Wang, X. and Lavrov, D. V. (2008). Seventeen new complete mtDNA sequences reveal extensive mitochondrial genome evolution within the Demospongiae. *PLoS One*, 3(7):e2723.

Wang, Y. and Sun, S. (2017). Complete mitochondrial genome of the jellyfish, *Rhopilema esculentum* Kishinouye 1891 (Cnidaria: Scyphozoa) and the phylogenetic relationship in the related species. *Mitochondrial DNA Part B*, 2(1):167–168.

Wolff, J. N., Camus, M. F., Clancy, D. J., and Dowling, D. K. (2016). Complete mitochondrial genome sequences of thirteen globally sourced strains of fruit fly (*Drosophila melanogaster*) form a powerful model for mitochondrial research. *Mitochondrial DNA Part A*, 27(6):4672–4674.

Yang, J., Zhu, Y., Tong, Y., Zhang, Z., Chen, L., Chen, S., Cao, Z., Liu, C., Xu, J., and Ma, X. (2009). The novel G10680A mutation is associated with complete penetrance of the LHON/T14484C family. *Mitochondrion*, 9(4):273–278.

Yang, Z. and Nielsen, R. (2000). Estimating synonymous and nonsynonymous substitution rates under realistic evolutionary models. *Mol. Biol. Evol.*, 17(1):32–43.

Zou, H., Zhang, J., Li, W., Wu, S., and Wang, G. (2012). Mitochondrial genome of the freshwater jellyfish *Craspedacusta sowerbyi* and phylogenetics of Medusozoa. *PLoS One*, 7(12):e51465.
